# 17 variants interaction of Wnt/β-catenin pathway associated with development of osteonecrosis of femoral head in Chinese Han population

**DOI:** 10.1038/s41598-024-57929-8

**Published:** 2024-03-27

**Authors:** Chuankai Shi, Xin Li, Yu Sun, Zhenwu Du, Guizhen Zhang, Zhenjia Che, Qingyu Li, Shiliang Song, Jing Guo, Haoyan Sun, Yang Song

**Affiliations:** 1 Medical Centre of Orthopedics, The Second Hospital of Jilin University, Ziqiang Street No.218, Nanguan District, Changchun City, 130041 Jilin Province China; 2 Gene Testing Centre of Changchun Tumor Hospital, Changchun City, 130012 Jilin Province China

**Keywords:** ONFH, Wnt/β-catenin pathway, Gene interaction, Lipid disorder, Coagulation abnormality, Genetics, Molecular biology, Risk factors

## Abstract

The genes of Wnt/β-catenin pathway may have potential roles in fat accumulation of Non-traumatic osteonecrosis of the femoral head (ONFH), but the effects of their variants in the pathway on ONFH development have been remained unclear. To explore the potential roles of the variants in the development of ONFH, we completed the investigation of the paired interactions as well as their related biological functions of 17 variants of GSK3β, LRP5, and FRP4 genes etc. in the pathway. The genotyping of the 17 variants were finished by MASS ARRAY PLATFORM in a 560 ONFH case–control system. The association of variants interactions with ONFH risk and clinical traits was evaluated by logistic regression analysis etc. and bioinformatics technology. The results showed that the genotype, allele frequency, and genetic models of Gsk3β rs334558 (G/A), SFRP4 rs1052981 (A/G), and LRP5 rs312778 (T/C) were significantly associated with the increased and decreased ONFH risk and clinical traits, respectively (P < 0.001–0.0002). Particularly, the paired interactions of six variants as well as eight variants also showed statistically increased and decreased ONFH risk, bilateral hip lesions risk and stage IV risk of ONFH, respectively (P < 0.044–0.004). Our results not only at the first time simultaneously showed exact serum lipid disorder and abnormal platelet function of ONFH in the same study system with the 17 variants polymorphisms of Wnt/β-catenin pathway but also shed light on the variants closely intervening the lipid disorder and abnormal coagulation of ONFH.

## Introduction

Non-traumatic osteonecrosis of the femoral head (ONFH) is a complex bone disorder related to genetic and environmental factors^1^. Although the pathogenesis of ONFH remains unclear, decreased osteogenesis together with increased adipogenesis is a common feature found in the bone marrow of osteonecrotic lesion since the transdifferentiation of BMSCs is regulated by the Wnt/β-catenin pathway^2–4^. It has been showed that critical genes of Wnt/β-catenin pathway play important roles in fat accumulation of the lesion bone marrow cavity of ONFH^5,6^, but it has rarely been reported that the effects of paired interactions of multiple variants in Wnt/β-catenin pathway genes on ONFH occurrence and development. The relevant reports suggest that chronic corticosteroid use and excessive alcohol consumption are the two main environmental risk factors of ONFH, and multiple genes are associated with ONFH risk^7,8^. In particular, some evidences revealed that the imbalance between osteogenesis and adipogenesis regulated by Wnt/β-catenin pathway played essential roles in the development of ONFH^9,10^. Moreover, much adipose tissue are often observed in the focus of ONFH so that lipid metabolism disorder has long been recognized as the core pathogenesis of ONFH^11,12^. In the view of the relationship between the multiple variants interaction of key genes in the Wnt/β-catenin pathway and the occurrence and development of ONFH has remained obscure, we selected the optimized 17 variants of GSK3β, LRP5, SFRP4, EMDR1, and LOC105375236 genes of the pathway and explored their possible molecular biological roles in the development of ONFH in a 560 subjects of ONFH-control study system.

## Methods

### Subjects

The study was conducted in accordance with the Declaration of Helsinki and approved by the Ethics Committee of the second hospital of Jilin University (No. 291, 2018 Research Approval), China. Written informed consent was obtained from all subjects involved in this study. A case–control study of 560 unrelated subjects includes in 260 of the patients with ONFH (age (years) 54.88 ± 11.61, male 184, female 76, BMI 24.52 ± 3.83) and 300 of healthy controls (age (years) 67.05 ± 8.62, male 131, female 169, BMI 24.37 ± 2.66). The ONFH patients who visited the Orthopedic Medical Centre of the Second Hospital of Jilin University from September 2015 to January 2019 were recruited based on the ONFH diagnosis criteria as described in reference^13^. Healthy controls were enrolled from September, 2018 to December, 2018 in the Health Physical Examination Center of the Second Hospital. Etiological classification (n (%)) of ONFH contains Alcohol-induced 118 (45.4), Steroid-induced 54 (20.8), and Idiopathic 88 (33.8), respectively. Hip lesions (n (%)) of ONFH contains unilateral hip lesion122 (46.9) and bilateraHip lesions 138 (53.1). Clinical stages (n (%)) of ONFH include Stage II 20 (7.7), Stage III 84 (32.3), and Stage IV 156 (60.0), respectively.

The detection of serum lipid levels and platelet indices were randomly selected 379 (ONFH123, Control 256) and 505(ONFH247, Control 248) from the subjects, respectively. The serum levels of triglyceride (TG), total cholesterol (TC)*,* high-density lipoprotein cholesterol (HDL-c), and low-density lipoprotein cholesterol (LDL-c) of the subjects were detected by BECKMAN COULTER AU680 automatic biochemical analyzer. And the platelet indices, including platelet (PLT), platelet crit (PCT), mean platelet volume (MPV), and platelet distribution width (PDW), were also measured.

### Genomic DNA extraction, variant selection, and genotyping

Approximately a 2 mL of venous blood from all the participants was collected after 10 h of fasting. According to the manufacturer’s protocol, the genomic DNA was extracted by using a genomic DNA extraction kit (DP318, TIANGEN, Beijing, China).The 17 variants of GSK3β, LRP5, SFRP4, EMDR1, and LOC105375236 were selected as the target variants of this study by Genome Variation Server 138 as described in references^13^. Simply, the database: http://gvs.gs.washington.edu/GVS138/ and related literature were used to select SNVs of the genes by analysing their population distribution in different countries, nationalities and regions, particularly in data obtained from an Asian population. The search scope was from the upstream 2000 bp to downstream 1000 bp of the five genes, respectively, including the promoter, 3-UTR, 5-UTR, intron, and exon region. The selection criteria of SNVs included in r^2^ = 0.8 or D′ = 1; Minority allele frequencies > 0.05. A list of the 17 variants and all PCR and sequencing primers designed using Sequenom Assay Design 3.1 software are shown in Supplementary Table 1. The genotyping of the17 variants were finished by using MASS ARRAY PLATFORM. The success rates of all variants genotype were generally high > 95%, respectively.

### Statistical analysis

The Hardy–Weinberg Equilibrium (HWE) test and the genotype and allele distribution in the ONFH and control groups were performed for each variant by the Shesis software platform (http://analysis.bio-x.cn/myAnalysis.php)^14^.

Multiple genetic models were used to assess the association of the 17 variants with ONFH risk by logistic regression analysis with adjusting for age and sex. The 2 × 2 gene interaction of multiplicative model among the 17 variants with ONFH risk, clinical stages, and hip lesions of ONFH were analyzed by logistical regression analyses, respectively. The generalized multifactor dimensionality reduction method (GMDR, version 0.7) were used to test high dimensional gene–gene interactions with ONFH risk.

The student’s t-test was used to analyze the differences of age, BMI, serum lipid levels, and platelet indices between ONFH and control groups. ONE-WAY ANOVA was used to finish the different analysis of the serum lipid levels, platelet indices, and the age at onset (years old) among the genotype of each variant. Measurement data are presented as mean ± standard deviation. The χ^2^ analysis was used to assess differences of gender, etiological classification, hip lesions and clinical stages among the genotypes of each variant. The above statistical analysis was performed using SPSS software (version 26, SPSS, Chicago, IL, USA). Statistical significance is defined as P < 0.05.

### Functional annotation and eQTLs analysis of the 17 variants in Wnt/β-catenin pathway

The HAPLOREG v4.1 database (https://pubs.broadinstitute.org/mammals/haploreg/haploreg.php) was employed for exploring functional annotations of the 17 variants. The genetic variants can be visualized along with protein binding information by searching this bioinformatical online tool. To examine the potential functions of the significant variants, we extracted gene expression data from the GTEX database (https://gtexportal.org/home/) to investigate the tissue-specific eQTL. The research data of GTEX have explored the differences in gene expression of different genotypes in various tissues and have indicated them with statistically significant P-values (P < 0.05).

## Results

### Association of the genotypes and allele frequencies of 17 variants in the Wnt/β-catenin pathway with ONFH risk

Genotype and allele frequency analysis in the 17 variants are represented in Table 1, Supplementary Table 2.

**Table 1:** Association of the genotypes and allele frequencies of 17 variants in Wnt/β-catenin pathway with ONFH risk.

Gene	Variant	Group	Genotype (n + %)			MAF	HWE	P^a^	Co-dominants (11 vs. 12 vs. 22)	Dominants (12 + 22 vs. 11)	Recessives (22 vs. 11 + 12)	Allele 2 vs. 1
			11	12	22				OR (95% CI) P^b^	OR (95% CI) P^b^	OR (95% CI) P^b^	OR (95% CI) P^b^
Gsk3β	rs2037547 (C/T)		CC	CT	TT							
		Control	255 (85.3)	44 (14.7)	0 (0.0)	0.074	0.385	0.059	0.701 (0.406–1.213)	0.776 (0.576–1.046)	–	0.863 (0.558–1.334)
		ONFH	230 (88.5)	27 (10.4)	3 (1.2)	0.063	0.071		0.204	0.096	–	0.505
	rs334558 (G/A)		GG	GA	AA							
		Control	85 (29.8)	150 (52.6)	50 (17.5)	0.439	0.280	0.001	1.622 (1.189–2.213)	1.377 (1.072–1.768)	1.360 (1.061–1.743)	1.259 (1.114–1.422)
		ONFH	45 (18.1)	133 (53.4)	71 (28.5)	0.552	0.249		0.002	0.012	0.015	0.0002
	rs3732361 (A/G)		AA	AG	GG							
		Control	110 (37.3)	131 (44.4)	54 (18.3)	0.405	0.184	0.491	1.284 (0.967–1.705)	1.229 (0.991–1.523)	1.121 (0.867–1.448)	1.091 (0.950–1.252)
		ONFH	85 (32.9)	118 (45.7)	55 (21.3)	0.442	0.256		0.084	0.060	0.383	0.217
LRP5	rs312778 (T/C)		TT	TC	CC							
		Control	238 (80.1)	58 (19.5)	1 (0.3)	0.101	0.333	0.346	0.539 (0.316–0.921)	0.735 (0.562–0.961)	–	0.803 (0.551–1.169)
		ONFH	217 (83.8)	42 (16.2)	0 (0.0)	0.081	0.389		0.024	0.024	–	0.251
LOC105375236	rs1721400 (C/T)		CC	CT	TT							
		Control	191 (64.3)	97 (32.7)	9 (3.0)	0.194	0.576	0.117	0.785 (0.541–1.140)	0.854 (0.687–1.061)	0.961 (0.546–1.692)	0.798 (0.615–1.034)
		ONFH	187 (72.2)	64 (24.7)	8 (3.1)	0.154	0.348		0.204	0.154	0.892	0.087
SFRP4	rs1052981 (A/G)		AA	AG	GG							
		Control	213 (71.7)	73 (24.6)	11 (3.7)	0.160	0.134	0.480	1.198 (0.834–1.720)	1.028 (0.825–1.282)	1.708 (1.041–2.805)	1.103 (0.848–1.435)
		ONFH	180 (70.6)	60 (23.5)	15 (5.9)	0.176	0.004		0.329	0.804	0.034	0.463

11, major allele homozygote; 12, allele heterozygote; 22, minor allele homozygote.

MAF, minor allele frequency; HWE, Hardy–Weinberg equilibrium.

^a^χ^2^ test (or Fisher exact test); ^b^logistic regression analyses.

The missing number of the 17 variants genotyping were different from 1 to 10 due to technological limitations of multiplex PCR. Generally, the genotype success rates of the17 variants were > 95%. The Gsk3β rs334558 (G/A) AA genotype (28.5%) and the A allele frequency (55.2%) in the ONFH patients were significantly higher than those of healthy controls, P = 0.001, OR (95% CI):1.259 (1.114–1.422), P = 0.0002, respectively. The genetic models of co-dominants, dominants, and recessives of rs334558 (G/A) all were associated with increased ONFH risk, OR (95% CI):1.622 (1.189–2.213), 1.377 (1.072–1.768), 1.360 (1.061–1.743), P = 0.002, 0.012, 0.015, respectively. The co-dominant and dominant model of LRP5 rs312778 (T/C) showed a protective effect on the decreased ONFH risk, 0.539 (0.316–0.921), P = 0.024, 0.735(0.562–0.961), P = 0.024, respectively, and the recessive model of SFRP4 rs1052981(A/G) revealed a significantly increased ONFH risk, 1.708 (1.041–2.805), P = 0.034. In addition, the polymorphism of Gsk3β rs2037547(C/T) and rs3732361(A/G), LOC105375236 rs1721400 (C/T) revealed a tendency of increased or decreased ONFH risk, respectively.

### Association of the genotypes of 17 variants in the Wnt/β-catenin pathway with the clinical traits of ONFH

The results were shown in Table 2, Supplementary Table 3. The age at onset of the AA carriers of Gsk3β rs3755557 (T/A) was significantly younger than that of TT carriers in ONFH patients (P = 0.001), as well as the AA carriers of steroid-induced ONFH was statistically higher than that of idiopathic ONFH (P = 0.026). Besides, the bilateral hip lesions (9.4%) of the CC carriers of SFRP4 rs2598116 (A/C) was significantly higher than that (1.6%) of unilateral hip lesions, P = 0.026. The female (26.2%) of the GG carriers of SFRP4rs1802073 (T/G) presented statistically higher than that of the male, P = 0.009.

**Table 2:** Association of the genotypes of 17 variants in Wnt/β-catenin pathway with the clinical traits.

Gene	Variant	genotype	Gender n (%)^a^		Age at onset (year)^b^	Etiological classification n (%)^a^			Hip lesions n (%)^a^		Clinical stages n (%)^a^		
			Male	Female		Alc	Ster	Idio	Unilateral	Bilateral	Stage II	Stage III	Stage IV
Gsk3β	rs2037547(C/T)	CC	273 (86.7)	212 (86.9)	48.88 ± 12.95	102 (86.4)	48 (88.9)	80 (90.9)	113 (92.6)	117 (84.8)	18 (90.0)	75 (89.3)	137 (87.8)
		CT	39 (12.4)	32 (13.1)	45.65 ± 11.09	13 (11.0)	6 (11.1)	8 (9.1)	7 (5.7)	20 (14.5)	2 (10.0)	8 (9.5)	17 (10.9)
		TT	3 (1.0)	0 (0.0)	55.00 ± 8.54	3 (2.5)	0 (0.0)	0 (0.0)	2 (1.6)	1 (0.7)	0 (0.0)	1 (1.2)	2 (1.3)
		P	0.304		0.344	0.612			0.058		0.982		
	rs3755557(T/A)	TT	226 (72.7)	154 (64.2)	50.43 ± 12.98	91 (77.8)	33 (62.3)	58 (65.9)	81 (67.5)	101 (73.2)	15 (78.9)	57 (68.7)	110 (70.5)
		TA	75 (24.1)	79 (32.9)	45.04 ± 11.39	22 (18.8)	17 (32.1)	30 (34.1)	34 (28.3)	35 (25.4)	4 (21.1)	21 (25.3)	44 (28.2)
		AA	10 (3.2)	7 (2.9)	38.21 ± 8.62	4 (3.4)	3 (5.7)	0 (0.0)	5 (4.2)	2 (1.4)	0 (0.0)	5 (6.0)	2 (1.3)
		P	0.074		0.001	0.026			0.324		0.310		
LRP5	rs312778(T/C)	TT	246 (78.8)	209 (85.7)	48.85 ± 12.57	94 (80.3)	47 (87.0)	76 (86.4)	104 (85.2)	113 (82.5)	18 (90.0)	71 (85.5)	128 (82.1)
		TC	65 (20.8)	35 (14.3)	47.17 ± 13.75	23 (19.7)	7 (13.0)	12 (13.6)	18 (14.8)	24 (17.5)	2 (10.0)	12 (14.5)	28 (17.9)
		CC	1 (0.3)	0 (0.0)	–	–	–	–	–	–	–	–	–
		P	0.058		0.453	0.392			0.547		0.576		
	rs3736228(C/T)	CC	196 (62.2)	135 (55.6)	48.04 ± 12.62	72 (61.0)	31 (57.4)	49 (55.7)	70 (57.4)	82 (59.4)	11 (55.0)	50 (59.5)	91 (58.3)
		CT	106 (33.7)	95 (39.1)	48.65 ± 13.09	41 (34.7)	22 (40.7)	35 (39.8)	48 (39.3)	50 (36.2)	7 (35.0)	32 (38.1)	59 (37.8)
		TT	13 (4.1)	13 (5.3)	58.88 ± 6.49	5 (4.2)	1 (1.9)	4 (4.5)	4 (3.3)	6 (4.3)	2 (10.0)	2 (2.4)	6 (3.8)
		P	0.273		0.065	0.830			0.817		0.611		
LOC105375236	rs1721400(C/T)	CC	215 (68.9)	163 (66.8)	48.45 ± 12.82	88 (75.2)	39 (72.2)	60 (68.2)	89 (73.0)	98 (71.5)	16 (80.0)	60 (71.4)	111 (71.6)
		CT	89 (28.5)	72 (29.5)	47.91 ± 12.45	26 (22.2)	14 (25.9)	24 (27.3)	29 (23.8)	35 (25.5)	4 (20.0)	20 (23.8)	40 (25.8)
		TT	8 (2.6)	9 (3.7)	58.25 ± 12.07	3 (2.6)	1 (1.9)	4 (4.5)	4 (3.3)	4 (2.9)	0 (0.0)	4 (4.8)	4 (2.6)
		P	0.704		0.093	0.758			0.938		0.852		
SFRP4	rs1802073(T/G)	TT	67 (21.6)	57 (24.1)	48.21 ± 12.93	25 (21.6)	9 (18.4)	21 (24.7)	21 (17.9)	34 (25.6)	4 (21.1)	16 (19.8)	35 (23.3)
		TG	191 (61.6)	118 (49.8)	48.67 ± 12.80	69 (59.5)	32 (65.3)	50 (58.8)	74 (63.2)	77 (57.9)	12 (63.2)	47 (58.0)	92 (61.3)
		GG	52 (16.8)	62 (26.2)	49.34 ± 12.99	22 (19.0)	8 (16.3)	14 (16.5)	22 (18.8)	22 (16.5)	3 (15.8)	18 (22.2)	23 (15.3)
		P	0.009		0.916	0.900			0.347		0.756		
	rs2598116(A/C)	AA	181 (57.5)	152 (62.0)	49.49 ± 12.87	72 (61.0)	35 (64.8)	52 (59.1)	77 (63.1)	82 (59.4)	13 (65.0)	56 (66.7)	90 (57.7)
		AC	119 (37.8)	74 (30.2)	47.54 ± 12.29	40 (33.9)	15 (27.8)	31 (35.2)	43 (35.2)	43 (31.2)	5 (25.0)	22 (26.2)	59 (37.8)
		CC	15 (4.8)	19 (7.8)	45.87 ± 14.07	6 (5.1)	4 (7.4)	5 (5.7)	2 (1.6)	13 (9.4)	2 (10.0)	6 (7.1)	7 (4.5)
		P	0.090		0.380	0.889			0.026		0.312		

Alc, alcohol-induced; Ster, steroid- induced; Idio, idiopathic.

^a^, χ^2^ test (or Fisher exact test); ^**b**^, one-way ANOVA (or Tukey test).

### Association of the paired gene–gene interactions of 17 variants in the Wnt/β-catenin pathway with ONFH risk, the hip lesions, and clinical stages of ONFH

**Figure 1:**
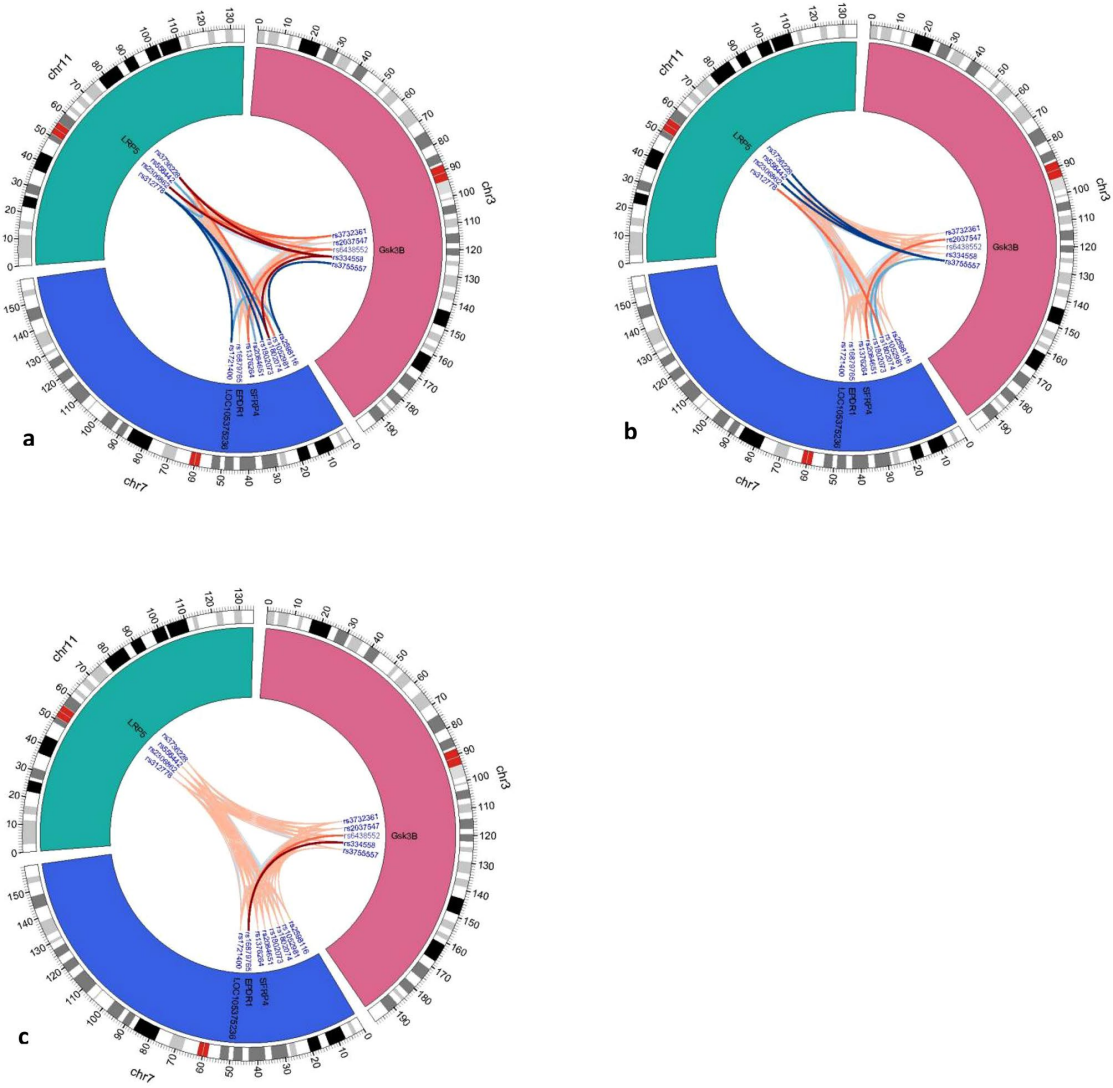
Association of the paired gene–gene interactions of 17 variants in Wnt/β-catenin pathway with the risk, hip lesions, and clinical stages of ONFH. The chromosomes are arranged end by end in a clockwise direction. The exterior of the circle is chromosome number and scale. The inner circle represents the gene where the variant is located. The innermost part is ID of the variant. The lines connecting two variants represents paired gene–gene interactions. The red lines represent interactions with OR > 1 while the blue lines represent interactions with OR < 1. In addition, the color shade and lines thickness indicate whether the interaction is statistical significance: the thinnest and lightest lines represent interactions with no statistical significance (P > 0.1), the middle ones represent interactions with 0.05 < P < 0.1, and the thickest and the darkest lines represent interactions with P < 0.05. (**a**) The paired gene–gene interactions of 17 variants in Wnt/β-catenin pathway with the ONFH risk. (**b**) The paired gene–gene interactions of 17 variants in Wnt/β-catenin pathway with the unilateral and bilateral hip lesions of ONFH. (**c**) The paired gene–gene interactions of 17 variants in Wnt/β-catenin pathway with the clinical stages of ONFH. Gsk3β, glycogen synthase kinase 3 beta; LRP5, LDL receptor related protein 5; SFRP4, secreted frizzled related protein 4; EPDR1, ependymin related 1; LOC105375236, uncharacterized LOC105375236, LncRNA.

The fifteen interactional variants in total were found to associate with ONFH risk (Fig. 1a, Supplementary Table 4), including the paired interaction between Gsk3β rs334558 and LRP5 rs2306862, Gsk3β rs334558 and LRP5 rs3736228, Gsk3β rs334558 and SFRP4 rs1802074, which were associated with significantly increased ONFH risk, OR (95% CI): 1.362 (1.036–1.789), 1.343 (1.031–1.751), 1.387 (1.057–1.821), P = 0.027, 0.029, 0.018, respectively. However, the paired interactions between Gsk3β rs3755557 and SFRP4 rs2598116, LRP5 rs312778 and LOC105375236 rs1721400, LRP5 rs312778 and SFRP4 rs1802073, LRP5 rs312778 and SFRP4 rs1802074 were associated with significantly decreased ONFH risk, OR (95% CI): 0.611 (0.382–0.976), 0.390 (0.175–0.868), 0.617 (0.386–0.987), 0.331 (0.157–0.696), P = 0.039, 0.021, 0.044, 0.004, respectively. Besides, the paired interactions of some variants also revealed a significant tendency of increased or decreased ONFH risk, respectively.

The six paid interactional variants showed significantly decreased bilateral hip lesions risk (Fig. 1b, Supplementary Table 5), including the interaction between Gsk3β rs3755557and LRP5 rs2306862, Gsk3β rs3755557 and LRP5 rs3736228, Gsk3β rs3755557 and LRP5 rs556442, OR (95% CI): 0.456 (0.222–0.939), 0.442 (0.216–0.906), 0.377 (0.191–0.746), P = 0.033, 0.026, 0.005, respectively. In addition, the paired interactions between Gsk3β rs2037547 and SFRP4 rs2084651, Gsk3β rs3755557 and SFRP4 rs1802073, Gsk3β rs3755557 and SFRP4 rs1802074, and LRP5 rs312778 and SFRP4 rs1802074 also revealed a significant tendency of increased or decreased bilateral lesions risk, respectively. The paired interactions between Gsk3β rs334558 and EPDR1 rs16879765 showed the significantly increased stage IV risk of ONFH, OR (95% CI): 2.270 (1.114–4.628), P = 0.024, whereas the interaction between Gsk3β rs6438552 and EPDR1 rs16879765 was marginally increased the stage IV risk (Fig. 1c, Supplementary Table 6).

### Associations of multidimensional interactions among 17 variants in the Wnt/β-catenin pathway with ONFH risk

The GMDR analysis revealed the identified four relatively best models with adjustment for covariates (age and sex), including in a one-locus model containing rs334558, a three-locus model containing rs334558- rs1052981- rs1376264, a five-locus model containing rs334558-rs6438552- rs556442- rs1802073-rs2084651, and a seven-locus model containing rs334558-rs6438552- rs556442- rs1721400- rs1052981-rs1802073- rs2084651 (training balanced accuracy: 0.5594, 0.6382, 0.7463, 0.8561, respectively, testing balanced accuracy: 0.5241, 0.5422, 0.5396, 0.5939, respectively, CV consistency: 9/10, 4/10, 6/10, 10/10, respectively). Although all the models mentioned above had only a trend towards significance (all P values = 0.0547), shown in Supplementary Table 7, the result reflected the potential effects of multiple gene interaction among 17 variants on ONFH risk.

### Lipid levels and platelet parameters between ONFH and Control groups and their association with the 17 variants polymorphism of Wnt/β-catenin pathway

**Figure 2:**
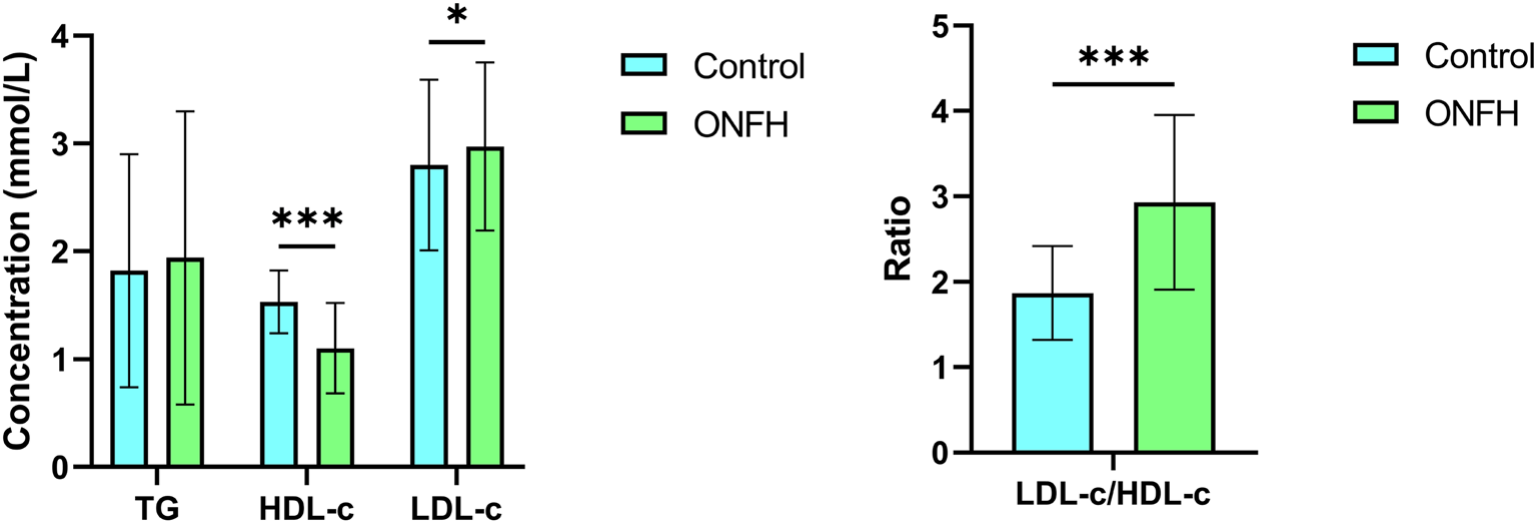
Analysis of serum lipid levels between ONFH and Control groups. *P < 0.05; ***P < 0.001.

**Figure 3:**
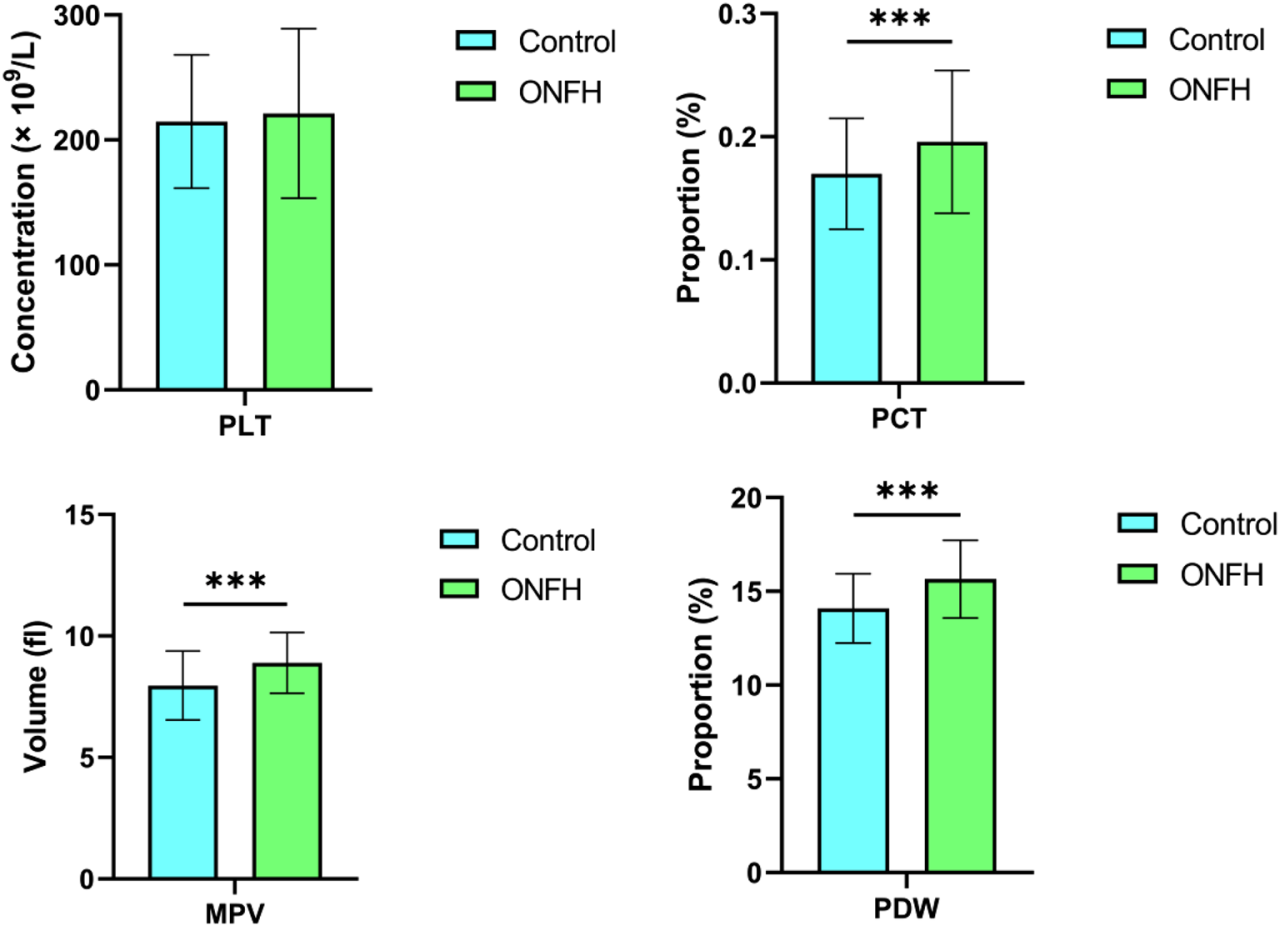
Analysis of PLT related parameters between ONFH and Control groups. ***P < 0.001.

Compared to control group, the decrease of HDL-c and the increace of LDL-c and LDL-c /HDL-c in ONFH patients showed statistical significance, P < 0.001, P = 0.048, and P < 0.001, respectively, see Fig. 2. The TC levels in the ONFH patients was lower than that of healthy controls because of a abnormal result for older age in healthy controls. Meanwhile, the PCT, MPV, and PDW levels of ONFH patients were also significantly increased (all P < 0.001), shown in Fig. 3**.**

Association of 17 variants genotypes in Wnt/β-catenin pathway with lipid levels and platelet parameters are shown in Table 3. The LDL-c /HDL-c of the TT carriers of Gsk3βs2037547(C/T) was significantly higher than that of the heterozygous CT carriers, P = 0.03, and the TC and HDL-c level in the AA carriers of Gsk3βrs334558(G/A) were significantly decreased, compared to those of the major homozygous GG carriers, P = 0.002, P = 0.005, respectively. And the TC level in the AA carriers and the LDLc/HDLc of the GG carrier of Gsk3βrs3732361 (A/G) were statistically higher than that of the heterozygous AG carriers, P = 0.033, P = 0.032, respectively. The TC level and LDL-c/HDL-c in the AA carrier of Gsk3β rs6438552(G/A) were decreased or increased, compared to that of the GG carrier and GA carriers, P = 0.040, 0.050, respectively. The HDLc level of the TT carrier in the SFRP4 rs1802073(T/G)showed significantly higher than that of heterozygous TG, P = 0.010.

**Table 3:** Association of the genotypes of 17 variants in Wnt/β-catenin pathway with lipid levels and platelet indices of ONFH.

Gene	Variant	Geno type	TG (mmol/L)	TC (mmol/L)	HDL-c (mmol/L)	LDL-c (mmol/L)	LDL-c/HDL-c	PLT (× 109/L)	PCT (%)	MPV (fl)	PDW (%)
Gsk3β	rs2037547(C/T)	CC	1.87 ± 1.18	5.11 ± 1.04	1.39 ± 0.40	2.89 ± 0.78	2.24 ± 0.89	218.36 ± 61.09	0.183 ± 0.053	8.41 ± 1.43	14.90 ± 2.09
		CT	1.82 ± 1.23	4.94 ± 1.03	1.44 ± 0.34	2.69 ± 0.85	1.97 ± 0.77	216.55 ± 61.72	0.180 ± 0.060	8.32 ± 1.36	14.44 ± 2.26
		TT	1.70 ± 0.17	4.54 ± 0.24	0.90 ± 0.06	2.99 ± 0.01	3.35 ± 0.23	191.00 ± 13.11	0.185 ± 0.004	9.63 ± 0.93	16.30 ± 0.26
		P	0.946	0.420	0.151	0.273	0.030	0.728	0.902	0.293	0.142
	rs334558(G/A)	GG	1.82 ± 1.06	5.40 ± 1.06	1.50 ± 0.36	2.99 ± 0.77	2.11 ± 0.77	225.75 ± 55.12	0.189 ± 0.053	8.44 ± 1.61	14.06 ± 2.17
		GA	1.89 ± 1.28	5.01 ± 1.07	1.38 ± 0.41	2.80 ± 0.83	2.19 ± 0.89	213.33 ± 61.27	0.179 ± 0.051	8.39 ± 1.32	15.09 ± 2.14
		AA	1.86 ± 1.08	4.90 ± 0.91	1.32 ± 0.37	2.83 ± 0.72	2.34 ± 0.93	217.85 ± 65.50	0.184 ± 0.056	8.55 ± 1.42	15.23 ± 1.76
		P	0.910	0.002	0.005	0.155	0.195	0.175	0.208	0.579	< 0.001
	rs3732361(A/G)	AA	1.92 ± 1.21	5.28 ± 1.10	1.39 ± 0.36	2.99 ± 0.82	2.31 ± 0.91	220.11 ± 63.55	0.185 ± 0.058	8.43 ± 1.45	14.45 ± 2.15
		AG	1.83 ± 1.22	4.98 ± 1.05	1.41 ± 0.40	2.77 ± 0.80	2.09 ± 0.78	214.61 ± 59.71	0.179 ± 0.049	8.38 ± 1.38	15.13 ± 2.13
		GG	1.85 ± 1.05	4.99 ± 0.88	1.35 ± 0.40	2.85 ± 0.70	2.35 ± 1.01	221.64 ± 60.66	0.186 ± 0.055	8.47 ± 1.45	14.87 ± 1.92
		P	0.808	0.033	0.556	0.067	0.032	0.535	0.382	0.841	0.006
	rs3755557(T/A)	TT	1.94 ± 1.17	5.11 ± 1.01	1.37 ± 0.37	2.88 ± 0.76	2.26 ± 0.91	218.45 ± 59.62	0.184 ± 0.053	8.44 ± 1.41	14.85 ± 1.97
		TA	1.71 ± 1.17	5.01 ± 1.09	1.45 ± 0.46	2.82 ± 0.86	2.11 ± 0.84	213.95 ± 65.30	0.177 ± 0.053	8.40 ± 1.42	14.94 ± 2.42
		AA	1.78 ± 1.42	5.20 ± 1.19	1.35 ± 0.24	2.90 ± 0.86	2.20 ± 0.71	234.94 ± 56.02	0.193 ± 0.065	8.16 ± 1.39	14.09 ± 1.76
		P	0.242	0.692	0.239	0.822	0.348	0.396	0.286	0.714	0.309
	rs6438552(G/A)	GG	1.91 ± 1.21	5.27 ± 1.10	1.40 ± 0.37	2.96 ± 0.81	2.28 ± 0.90	220.97 ± 63.65	0.186 ± 0.058	8.43 ± 1.47	14.50 ± 2.15
		GA	1.80 ± 1.17	5.00 ± 1.03	1.42 ± 0.41	2.78 ± 0.80	2.09 ± 0.79	213.61 ± 59.47	0.178 ± 0.050	8.37 ± 1.39	15.05 ± 2.17
		AA	1.87 ± 1.07	4.96 ± 0.86	1.34 ± 0.40	2.83 ± 0.70	2.35 ± 1.03	220.02 ± 61.22	0.186 ± 0.055	8.54 ± 1.44	14.97 ± 1.86
		P	0.709	0.040	0.312	0.148	0.050	0.445	0.259	0.615	0.029
LRP5	rs2306862(C/T)	CC	1.89 ± 1.13	5.09 ± 0.97	1.40 ± 0.41	2.86 ± 0.76	2.21 ± 0.89	219.66 ± 58.32	0.183 ± 0.053	8.38 ± 1.40	14.84 ± 2.12
		CT	1.80 ± 1.28	5.07 ± 1.17	1.39 ± 0.35	2.89 ± 0.86	2.22 ± 0.89	211.99 ± 60.14	0.180 ± 0.054	8.46 ± 1.45	14.94 ± 2.10
		TT	1.91 ± 1.07	5.08 ± 0.96	1.35 ± 0.41	2.69 ± 0.70	2.12 ± 0.78	236.79 ± 91.97	0.197 ± 0.062	8.53 ± 1.54	14.25 ± 1.99
		P	0.772	0.988	0.829	0.609	0.901	0.126	0.345	0.795	0.327
	rs312778(T/C)	TT	1.82 ± 1.14	5.10 ± 1.03	1.40 ± 0.40	2.86 ± 0.79	2.22 ± 0.92	217.01 ± 60.68	0.184 ± 0.055	8.48 ± 1.44	14.85 ± 2.16
		TC	2.05 ± 1.35	5.09 ± 1.05	1.39 ± 0.33	2.92 ± 0.78	2.20 ± 0.67	224.25 ± 62.03	0.179 ± 0.048	8.09 ± 1.27	14.85 ± 1.89
		CC	–	–	–	–	–	–	–	–	–
		P	0.203	0.933	0.807	0.534	0.849	0.307	0.450	0.018	0.996
	rs3736228(C/T)	CC	1.90 ± 1.15	5.10 ± 0.98	1.41 ± 0.41	2.86 ± 0.77	2.21 ± 0.90	217.12 ± 56.65	0.181 ± 0.052	8.39 ± 1.41	14.88 ± 2.09
		CT	1.81 ± 1.25	5.04 ± 1.16	1.36 ± 0.34	2.88 ± 0.84	2.24 ± 0.87	216.07 ± 62.87	0.184 ± 0.055	8.49 ± 1.44	14.93 ± 2.13
		TT	1.84 ± 1.10	5.09 ± 0.84	1.37 ± 0.40	2.73 ± 0.63	2.14 ± 0.78	238.42 ± 90.85	0.194 ± 0.063	8.27 ± 1.46	14.04 ± 2.06
		P	0.764	0.861	0.599	0.744	0.873	0.232	0.495	0.680	0.142
	rs556442(A/G)	AA	1.89 ± 1.14	5.10 ± 1.02	1.41 ± 0.43	2.87 ± 0.80	2.22 ± 0.91	217.06 ± 58.58	0.182 ± 0.053	8.44 ± 1.45	14.81 ± 2.18
		AG	1.75 ± 1.15	5.06 ± 1.11	1.40 ± 0.37	2.87 ± 0.80	2.20 ± 0.87	215.82 ± 60.39	0.183 ± 0.054	8.45 ± 1.42	14.98 ± 2.07
		GG	2.20 ± 1.43	5.13 ± 0.93	1.34 ± 0.33	2.81 ± 0.72	2.20 ± 0.76	232.16 ± 75.54	0.186 ± 0.057	8.10 ± 1.30	14.31 ± 1.90
		P	0.091	0.880	0.671	0.898	0.988	0.242	0.890	0.291	0.147
EPDR1	rs16879765(C/T)	CC	1.90 ± 1.23	5.13 ± 1.04	1.40 ± 0.40	2.88 ± 0.79	2.23 ± 0.90	219.51 ± 61.86	0.184 ± 0.053	8.41 ± 1.39	14.82 ± 2.17
		CT	1.71 ± 0.88	4.88 ± 1.04	1.38 ± 0.34	2.74 ± 0.79	2.11 ± 0.80	210.99 ± 57.82	0.178 ± 0.055	8.47 ± 1.58	14.93 ± 1.86
		TT	1.67 ± 0.92	4.88 ± 0.14	1.31 ± 0.17	2.80 ± 0.26	2.18 ± 0.51	212.00 ± 32.36	0.166 ± 0.025	7.70 ± 0.56	15.58 ± 1.53
		P	0.503	0.190	0.858	0.415	0.609	0.479	0.487	0.106	0.718
LOC105375236	rs1721400(C/T)	CC	1.89 ± 1.23	5.10 ± 1.05	1.38 ± 0.41	2.88 ± 0.77	2.26 ± 0.91	222.81 ± 61.80	0.186 ± 0.056	8.38 ± 1.4	14.84 ± 2.09
		CT	1.75 ± 0.94	5.04 ± 0.98	1.41 ± 0.34	2.83 ± 0.83	2.12 ± 0.81	208.23 ± 58.59	0.176 ± 0.047	8.47 ± 1.5	14.78 ± 2.08
		TT	2.46 ± 1.95	5.17 ± 1.37	1.37 ± 0.39	2.90 ± 0.89	2.28 ± 1.00	201.43 ± 59.26	0.178 ± 0.064	8.71 ± 1.1	15.45 ± 2.91
		P	0.296	0.855	0.833	0.861	0.407	0.032	0.223	0.575	0.488
SFRP4	rs1052981(A/G)	AA	1.93 ± 1.24	5.10 ± 1.05	1.39 ± 0.38	2.85 ± 0.80	2.23 ± 0.94	218.47 ± 61.77	0.183 ± 0.054	8.44 ± 1.43	14.82 ± 2.19
		AG	1.71 ± 1.02	5.02 ± 1.03	1.41 ± 0.46	2.83 ± 0.76	2.14 ± 0.72	217.68 ± 57.33	0.181 ± 0.048	8.26 ± 1.37	14.91 ± 1.84
		GG	1.56 ± 0.81	5.08 ± 0.98	1.36 ± 0.28	3.00 ± 0.73	2.32 ± 0.84	212.74 ± 66.79	0.183 ± 0.073	8.50 ± 1.53	14.85 ± 2.02
		P	0.164	0.830	0.836	0.738	0.643	0.907	0.901	0.443	0.911
	rs1376264(G/A)	GG	1.95 ± 1.26	5.11 ± 0.98	1.42 ± 0.41	2.84 ± 0.77	2.18 ± 0.92	218.26 ± 61.91	0.184 ± 0.054	8.46 ± 1.42	14.88 ± 2.18
		GA	1.71 ± 1.03	5.04 ± 1.20	1.35 ± 0.36	2.86 ± 0.85	2.24 ± 0.81	215.76 ± 58.75	0.178 ± 0.052	8.28 ± 1.32	14.83 ± 1.94
		AA	1.73 ± 0.85	5.10 ± 0.95	1.36 ± 0.30	3.05 ± 0.79	2.36 ± 0.83	227.39 ± 59.86	0.190 ± 0.062	8.38 ± 1.69	14.42 ± 2.18
		P	0.177	0.817	0.339	0.505	0.611	0.647	0.383	0.470	0.543
	rs1802073(T/G)	TT	1.76 ± 1.07	5.22 ± 1.19	1.51 ± 0.52	2.90 ± 0.89	2.09 ± 0.86	213.62 ± 58.14	0.180 ± 0.054	8.50 ± 1.51	14.71 ± 2.47
		TG	1.85 ± 1.14	4.99 ± 0.94	1.34 ± 0.33	2.85 ± 0.75	2.27 ± 0.86	214.23 ± 58.59	0.179 ± 0.052	8.35 ± 1.40	14.82 ± 2.05
		GG	1.98 ± 1.37	5.18 ± 1.14	1.43 ± 0.38	2.84 ± 0.81	2.17 ± 0.96	226.39 ± 62.45	0.192 ± 0.053	8.53 ± 1.43	15.02 ± 1.85
		P	0.507	0.162	0.010	0.857	0.293	0.170	0.098	0.436	0.536
	rs2084651(C/G)	CC	1.82 ± 1.06	5.07 ± 1.02	1.44 ± 0.46	2.85 ± 0.78	2.17 ± 0.92	212.31 ± 58.62	0.180 ± 0.051	8.45 ± 1.45	14.88 ± 2.21
		CG	1.88 ± 1.29	5.03 ± 1.05	1.37 ± 0.37	2.81 ± 0.80	2.21 ± 0.89	220.69 ± 61.02	0.185 ± 0.055	8.44 ± 1.40	14.78 ± 2.11
		GG	1.88 ± 1.02	5.25 ± 1.06	1.40 ± 0.32	2.98 ± 0.75	2.25 ± 0.78	221.32 ± 63.72	0.182 ± 0.055	8.25 ± 1.42	14.93 ± 1.89
		P	0.896	0.304	0.335	0.296	0.830	0.329	0.629	0.490	0.806
	rs2598116(A/C)	AA	1.89 ± 1.19	5.12 ± 1.04	1.38 ± 0.34	2.90 ± 0.77	2.24 ± 0.84	222.03 ± 62.16	0.185 ± 0.054	8.38 ± 1.43	14.83 ± 1.93
		AC	1.86 ± 1.20	5.01 ± 1.01	1.40 ± 0.47	2.78 ± 0.80	2.18 ± 0.95	210.79 ± 57.64	0.179 ± 0.053	8.49 ± 1.35	14.95 ± 2.41
		CC	1.61 ± 0.85	5.21 ± 1.23	1.54 ± 0.44	2.91 ± 0.98	2.06 ± 0.97	215.71 ± 64.53	0.178 ± 0.056	8.39 ± 1.71	14.47 ± 2.07
		P	0.616	0.564	0.211	0.387	0.586	0.154	0.510	0.696	0.490
	rs1802074(C/T)	CC	1.87 ± 1.21	5.13 ± 0.99	1.41 ± 0.42	2.88 ± 0.78	2.23 ± 0.93	218.64 ± 60.68	0.185 ± 0.052	8.48 ± 1.41	14.99 ± 2.04
		CT	1.89 ± 1.18	5.04 ± 1.11	1.38 ± 0.36	2.86 ± 0.79	2.20 ± 0.83	215.76 ± 61.97	0.177 ± 0.054	8.23 ± 1.37	14.68 ± 2.21
		TT	1.66 ± 0.69	5.18 ± 1.02	1.34 ± 0.32	2.98 ± 0.82	2.33 ± 0.75	222.22 ± 59.78	0.196 ± 0.058	8.90 ± 1.76	14.44 ± 2.29
		P	0.769	0.655	0.703	0.840	0.845	0.827	0.153	0.040	0.188

TG, triglyceride(0.56–1.71 mmol/L)^#^; TC, total cholesterol (2.90–5.17 mmol/L)^#^; HDL-c, high-density lipoprotein cholesterol (0.90–1.68 mmol/L)^#^; LDL-c, low-density lipoprotein cholesterol (2.70–3.40 mmol/L)^#^.PLT, platelets (125–350/L × 10^9^)^#^; PCT, Plateletcrit (0.108–0.282%)^#^; MPV, Mean platelet volume (7.0–11.0↑fl)^#^; PDW, platelet distribution width (9.0–17.0%)^#^. ^#^Reference value; P: one-way ANOVA (or Tukey test).

Moreover, the PLT of the TT carriers of LOC105375236 rs1721400(C/T) was significantly decreased, compared to that of the CC carriers, P = 0.032, while the PDW(%)in the AA carries of Gsk3βrs334558(G/A) was obviously lower than that of the GG carrier, P < 0.001. The PDW(%)in the AA carriers of Gsk3βrs3732361 (A/G) and GG carriers of Gsk3βrs6438552(G/A) were also significantly lower than that of the heterozygous carriers, P = 0.006, P = 0.029, respectively. And the MPV(fl) in the TT carriers of LRP5rs312778(T/C) and in the TT carries of SFRP4 rs1802074(C/T) all revealed statistically higher than that of heterozygous TC and CT carriers, P = 0.018, P = 0.040, respectively.

### Potential regulatory role and eQTL gene expression analysis in the 17 variants of Wnt/β-catenin pathway

**Figure 4:**
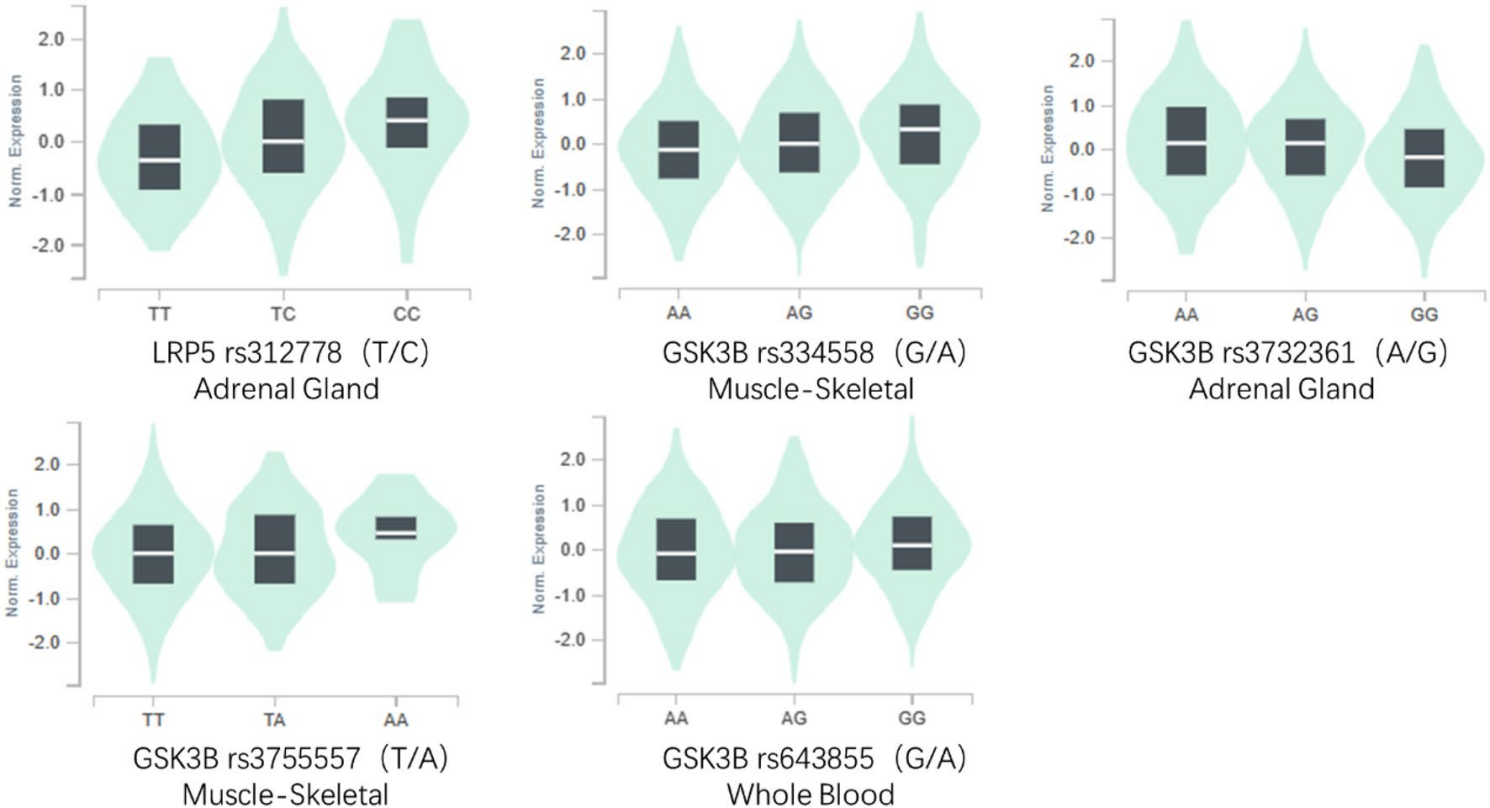
The effects of genotypes of eQTLs variants in Wnt/β-catenin pathway on gene expression in different tissues.

To explore the functional effects and their potential regulatory roles of the 17 variants, we performed a functional prediction of statistically significant variants by using the HaploReg database. The results exhibited that rs334558, rs3732361, rs3755557 and rs6438552 of Gsk3β and rs312778 of LRP5 revealed their potential biological functions, as shown in Table 4. The five variants all are located in 5 prime UTR of Gsk3β and LRP5 gene, respectively. As motif variants, they play a switch role in controlling transcription factor binding to the gene and affecting its transcription and mRNA expression. Besides, the variants, also as promoter histone marks and DNAse function, exert multiple effects. The eQTLs analysis results also show that the 5 variants were closely associated with the modulation of gene expression because they are identified as eQTLs in different tissues expressions with significant p-values. The significant effects of different genotypes on the gene expressions in the above five eQTs variants are shown in Fig. 4. The expression of minor homozygous GG genetype of eQTLrs 334558 of Gsk3β is the highest among three genotypes in muscle skeletal, while the major homozygous AA genotype in eQTLrs 2732361 of Gsk3β has the highest expressions in subcutaneous adipose tissue. Moreover, the expressions of minor homozygous AA and heterozygous TA genetypes of eQTLrs 3755557 of Gsk3β are the higher than that of major homozygous TT genotype in muscle skeletal. And the minor homozygous GG genetype of eQTLrs 6438552 of Gsk3β and the minor homozygous CC genetype of eQTLrs312778 of LRP5 also reveal the highest expressions among three genotypes in whole blood and adrenal grand, respectively.

**Table 4:** Variants functional annotation and eQTL analysis of significance in the Wnt/β- catenin pathway.

SNP	Gene	Ref	Alt	SNP functional annotation	Tissue	P-value	NES
rs334558	Gsk3β	A	G	Promoter histone marks, DNAse, Proteins bound, Motifs changed, Selected eQTL hits	Muscle–Skeletal	2.60E−08	0.11
rs3732361	Gsk3β	A	G	Motifs changed, GRASP QTL hits, Selected eQTL hits	Adipose–Subcutaneous	1.00E−14	− 0.22
rs3755557	Gsk3β	T	A	Promoter histone marks, DNAse, Motifs changed	Muscle–Skeletal	1.30E−04	0.11
rs6438552	Gsk3β	A	G	Promoter histone marks, Motifs changed, GRASP QTL hits, Selected eQTL hits	Whole Blood	1.20E−04	0.065
rs312778	LRP5	T	C	Promoter histone marks, DNAse, Proteins bound, GRASP QTL hits, Selected eQTL hits	Adrenal Gland	1.50E−06	0.3

Ref, reference; Alt, alternation; eQTL, expression quantitative trait locus; NES, normalized effect size.

## Discussion

The multiple functions of Wnt signaling pathway include mitogen stimulation, cell fate determination, and cell differentiation^15^. Of them, Wnt/β-catenin pathway affects bone formation and remodeling processes^16,17^. The human Wnt family is composed of 19 highly conserved genes that regulate gene expression, cell behavior, and cell polarity. GSK3β located in 3q13.33 is a protein coding gene, and it is the initiation factor of the Wnt/β-catenin pathway and as a downstream protein directly interacting with AKT in the PI3K/Akt signaling pathway^18^. Considering the critical roles of GSK3β in bone metabolism, we focused on the effects of its five variants on the development of ONFH. Our results showed that the AA genotype and the A allele frequency of Gsk3βrs334558 (G/A) were significantly associated with the increased ONFH risk, as well as the genetic models of co-dominants, dominants, and recessives of the variant all were related to the increased risk. Moreover, the paired interaction of Gsk3β rs334558 with LRP5 rs2306862, with LRP5 rs3736228, and with SFRP4 rs1802074 also showed a significantly increased ONFH risk, respectively, whereas the paired interactions between Gsk3β rs3755557 and SFRP4 rs2598116 revealed a decreased ONFH risk. ONFH is a complex disease involved in genes and environmental risk factors. Even if the identified variants from genome-wide association study(GWAS) often show only modest effects on the disease risk, leading to the “missing heritability”. The gene–gene interactions (epistasis) analysis can solve a part of this “missingness” to thereby elucidating their effect on complex diseases^19^. The paired interactions among the variants of GSK3β gene itself or with other gene variants strongly revealed the contribution of epistatic effects to ONFH risk, that is, recovered the missing heritability of ONFH. GWASs has been reported that the “missingness” is attributed to genetic heterogeneity, epistasis (gene–gene interaction), and gene environment interaction^20,21^. Moreover, it has been investigated that epistasis could drive the evolution of recombination frequencies among genes on the same chromosome, thereby altering gene order^22^. The gene–gene interactions include synthetic, suppressive, and epistatic types. When Gsk3β rs334558, together with the other multiple variants from residing in Wnt signaling pathway, may produce a new trait with synthetic and epistatic types, increased or decreased ONFH risk^23^.

Our results enough revealed that Gsk3β rs334558*,* as an essential variant, involved in the development of ONFH. What is the possible mechanism of this variant leading to ONFH? First, rs334558 is a 5 prime UTR variant, as motif variant, it is a switch that controls transcription factor binding to Gsk3β gene and affecting its transcription. Second, rs334558, as a eQTL variant, may play potential regulatory role in Gsk3β gene expression. Besides, the variant, also as a promoter histone mark and DNAse function, exerts multiple effects. In the HaploReg database, we performed a functional prediction of the significant variants. And the results showed rs334558, rs3732361, rs3755557 and rs6438552 of Gsk3β all exhibited potential biological functions in gene modulation. The variants were identified in different tissues expression with significant p-values.Using the GTEx database,we also find that the four variants regulate the functional importance of Gsk3β gene expression as eQTLs. The variants potential functions of GSK3β discovered by the databases analysis may explain their molecular mechanism during the development of ONFH.

SFRP4 acts as soluble modulator of Wnt signaling and exerts critical roles in regulating bone formation and resorption as the main receptor for Wnt^24–26^. In our evaluation results of the six variants of SFRP4, the rs1802074 showed the increased ONFH risk by the epistasis with Gsk3β rs334558, and the increased bilateral hip lesions risk in the CC carriers of SFRP4 rs2598116 (A/C). The recessives model of SFRP4 rs1052981 was reported to relate to increased ONFH risk and knee OA in women^27^. Previous results had also revealed a significant association between SFRP4 rs1802073 and BMD in Danish and Belgian men^28^, and the variant also connected with elevated serum lipid levels after acitretin treatment^29^. Our results also showed that the female percentage of minor homozygous GG of the variant was statistically higher than that of the male. The close association of the SFRP4 variants with ONFH risk and its clinical traits indicated their potential biological roles in the development of ONFH.

LRP5 also acts as a co-receptor with Fz protein family members for transducing signals by Wnt proteins. It plays a key role in skeletal homeostasis, and the many diseases related bone density are caused by mutations in this gene. This study involved the four variants of LRP5, and both the co-dominant and dominant model of LRP5 rs312778 (T/C) showed a protective role in decreased ONFH risk, while the LRP5 rs2306862 and rs3736228 connected with the increased ONFH risk by the paired interaction with Gsk3β rs334558. With using the HaploReg database, the LRP5 rs312778 was also found to played the roles of promoter histone marks, DNAse, and proteins bound. By GTEx database, the variant was further identified as an eQTL of regulating gene expression, exhibited its potential biological roles in the development of ONFH. As Wnt co-receptor, LRP5 has been identified as a crucial protein for mechanical signaling in bone, and the modifiers of LRP5 activity play important roles in mediating signaling efficiency^30^. LRP5 gene has been focused on GWAS and has confirmed a close relationship of its genotypes with osteoporosis^31,32^. Therefore, the associations between the variants of LRP5 gene and BMD or osteoporotic fractures have been extensively explored. Rs4988300 and rs634008 of LRP5 gene also associated with bone phenotypes in the elderly with OP or fractures^33^.To our knowledge, the association of the four variants of LRP5 with the development of ONFH was for the first time reported in this study.

LOC105375236 is a lncRNA located in 7p14.1, and the rs1721400(C/T), previously thought to be a variant of GSK3β gene, and now belonging to LOC105375236, is a genetic downstream transcript variant. In a polygenic inheritance GWAS study, the intergenic variant rs1524058 locates between LOC105375236 and STARD3NLwas identified as one of pleiotropic variants related to all four traits^34^. In our present study, the one of best model composed of 7 variants containing rs1721400 (C/T) in GMDR analysis connected with ONFH risk tendency. The protein encoded by EPDR1 gene is a type II transmembrane protein similar to two families of cell adhesion molecules^35^. EPDR1was reported to be closely related to the developmental processes of various tumors^36–38^^.^ Our results revealed that the paired interactions between EPDR1 rs16879765(C/T) and Gsk3β rs334558 significantly increased stage IV risk of ONFH. And we have also introduced the GMDR approach to our study and the results revealed four best models related to ONFH risk were set up. Even though failed to reach significance (all P-values = 0.0547), GMDR analysis is a useful tool to predict ONFH risk in expanded samples.

Our results also revealed that the age at onset of AA carriers of Gsk3β rs3755557 (T/A) was significantly younger than that of TT carriers, as well as the proportion of steroid-induced ONFH of the AA carriers was statistically higher than that of idiopathic ONFH. Moreover, the paid interaction between Gsk3β rs3755557 and LRP5 rs2306862, LRP5 rs3736228, and LRP5 rs556442 showed significantly decreased bilateral hip lesions risk, while the interactions between Gsk3β rs334558 and EPDR1 rs16879765 revealed the increased stage IV risk of ONFH, which indicate that the interaction of the variants strongly involved in the development of ONFH.

Fat accumulation is often observed in the lesion bone marrow cavity of ONFH in the animal experiments as well as the clinical studies^39,40^. Here, we clearly showed not only the increase of serum LDLc, LDLc/HDLc and the decrease of HDLc but also the rise of PCT, MPV, and PDW parameters of ONFH patients, which at the first time provided forceful evidence that there were simultaneous lipid metabolic disorders and coagulation disturbance in the same research system of focusing on the roles of 17 variants in Wnt/β-catenin pathway in ONFH development. Fat embolism and coagulation disorders are considered the two possible causes of interruption of blood supply in ONFH^41^. It is well-known that platelet indices are closely associated with platelet function. MPV indicates early platelet activation, and the increased MPV shows that the release of thromboxane A2 from large platelets is more than from smaller ones^42^. PCT (PLT × MPV) reflects the volume of blood occupied by platelets. As an index of the size uniformity of PLTs, PDW is the marker of platelet activity, and the increased PDW level are associated with abnormal thrombosis^43^. Especially, the variants in Wnt /β- catenin pathway were significantly involved in the lipid metabolic disorders and abnormal coagulation of ONFH by different genotypes and genetic model, which strongly indicates that the possible roles of the variants in the hyperlipidemia as well as the hypercoagulation of ONFH. The pathological features of ONFH are affected by impaired MSCs differentiation, and the Wnt/β-catenin pathway plays a vital role in the lineage specification during MSCs differentiation.^.^ Some studies also show steroid- and alcohol-induced ONFH from the inhibition of MSCs pathway. Our previous works found that a molecular axis driven by circRNA CDR1as- miR-7-5p-WNT5B and a molecular axis driven by lncRNA HOTAIR- miRNA-217-DKK1 played essential roles in the transdifferentiation between osteogenesi and adipogenesis of BMSCs in ONFH^44,45^. Both the molecular axes vigorously promoted the adipogenesis and inhibited the osteogenesis of the BMSCs. Especially significant, their ending protein molecules, WNT5B and DKK1, respectively, also are as key proteins of Wnt/β-catenin pathway, which presented the evidence of the abnormal molecular damage network driven by non-coding RNAs and eventually collected the Wnt/β-catenin pathway, elucidated the molecular mechanism of ONFH fat accumulation and the molecular targets for early prevention and treatment of ONFH. The evidences were also highly consistent with the results of multiple variants in Wnt/β-catenin pathway involving in the development of ONFH. Therefore, the variants in Wnt/β-catenin pathway facilitate specific biological events, the lipid and coagulation disorder, the differentiation of BMSCs, and the abnormal molecular damage network leading to the development of ONFH.

Our study have several limitations. First, the samples size of 560 case–control was smaller, which may have limited our statistical power to detect slight differences between groups, and further expanded samples investigation will improve our statistical results. Second, there was a difference in age and gender between ONFH and control group due to the older age and more female of control group, which may also affect the statistical results because of age and gender as covariates of analysis. Future research will overcome the bias through precise selection of enrolling participants. Third, although the serum lipid levels and platelet parameters between ONFH and control group revealed statistical significance, the actual difference values between two groups were smaller. A accurate grouping or stratification of enrolled ONFH patients in future research may improve such studies. Fourth, the molecular biological roles of these variants in the development of ONFH remain to be deeply identified by multiple complex verification experiment.

Fifth, although our study revealed the effects of Wnt pathway variants on ONFH development involving in lipid metabolism disorders and platelet parameters changes, the exact roles and its mechanisms of Wnt /β-catenin pathway in lipid metabolism and platelet regulation of ONFH need to be in-depth explored in future study..

## Conclusions

Our results at the first time revealed that the genotypes, allele frequency, and genetic models of Gsk3β rs334558, SFRP4 rs1052981, and LRP5 rs312778 in Wnt/β-catenin pathway were associated with increased and decreased ONFH risk, respectively, and the paired interactions of six variants and eight variants in the pathway were statistically connected with the increased or decreased ONFH risk, respectively. The interactions of six paired variants were also related to the decreased risk of bilateral hip lesions of ONFH, while the paired interactions of two variants closely connected with the increased stage IV risk of ONFH. In particular, we showed that the crucial variants in Wnt/β-catenin pathway were involved in the lipid disorder and abnormal coagulation of ONFH and played significant roles during the occurrence and development of ONFH.

### Supplementary Information

Supplementary Tables.

## Data Availability

All data used to support the findings of this study are included within the article/Supplementary Tables, and they are available upon request from the corresponding author.
