# Supplementary material for: 17 variants interaction of Wnt/β-catenin pathway associated with development of osteonecrosis of femoral head in Chinese Han population
Source: Sci Rep. 2024 Mar 27;14:7301. doi: 10.1038/s41598-024-57929-8 (PMC10973331; doi:10.1038/s41598-024-57929-8)
Supplement: Supplementary file 1 — Supplementary Tables. [file 41598_2024_57929_MOESM1_ESM.zip › Supplementary Tables/Supplementary Table 1.docx]

**Supplementary Table 1. Basic information and primers for PCR and sequencing of 17 variants in Wnt/β-catenin pathway**

| Gene | Chr | Variant ID | Allele (+/-) | Position（GRCh38） | Function | PCR primer | Sequencing primer |
| --- | --- | --- | --- | --- | --- | --- | --- |
| Gsk3β | 3q13.33 | rs2037547 | C/T (+) | NC_000003.12:g.119825768C>T | 3' UTR | 5'ACGTTGGATGCTTGAGAGGACATTGTAGTT3' | AAAATATGAAAAATAAACCAGTAGAC |
|  |  |  |  |  |  | 5'ACGTTGGATGGGCTATTTCTGCAAGCTCAA3' | AAAATATGAAAAATAAACCAGTAGAT |
|  |  | rs334558 | G/A (+) | NC_000003.12:g.120094435G>A | Promoter | 5'ACGTTGGATGAGACAGCGCTCCTCACACAG3' | TTCGCCCGGGTCAGGA |
|  |  |  |  |  |  | 5'ACGTTGGATGTTCCTTTGTCACTTGGCCCG3' | TTCGCCCGGGTCAGGG |
|  |  | rs3732361 | A/G (+) | NC_000003.12:g.119823450A>G | 3' UTR | 5'ACGTTGGATGCCCAAATGAGAGAGTGACAG3' | TGACGTATCAAAACCTGATACTATTAA |
|  |  |  |  |  |  | 5'ACGTTGGATGAGCATTTTAGACCACTGACG3' | TGACGTATCAAAACCTGATACTATTAG |
|  |  | rs3755557 | T/A (+) | NC_000003.12:g.120096110T>A | Promoter | 5'ACGTTGGATGCCTGCAGAGTCATCTCTTTC3' | ATCTGATCAAATATAGGTCCTTTA |
|  |  |  |  |  |  | 5'ACGTTGGATGGTTTCAAAGCAAGAGCCAGG3' | ATCTGATCAAATATAGGTCCTTTT |
|  |  | rs6438552 | G/A (+) | NC_000003.12:g.119912967G>A | Intron | 5'ACGTTGGATGCTTTTTTGCAGAGCAAGGTG3' | AGAAAATGTAAACTGTAACTATCTCTA |
|  |  |  |  |  |  | 5'ACGTTGGATGGATTCACATCATTTGAATCAT3' | AGAAAATGTAAACTGTAACTATCTCTG |
| LRP5 | 11q13.2 | rs2306862 | C/T (+) | NC_000011.10:g.68410042C>T | Synonymous | 5'ACGTTGGATGAAGAACCTCTACTGGGCCGA3' | cccctCGCGCCACTTCGATTCTA |
|  |  |  |  |  |  | 5'ACGTTGGATGTTGTCCAAGTCCCTCCACAC3' | cccctCGCGCCACTTCGATTCTG |
|  |  | rs312778 | T/C (+) | NC_000011.10:g.68340864T>C | Intron | 5'ACGTTGGATGTTACCCAGAGACTTGTCTCC3' | AGCACCAGCGGAGACA |
|  |  |  |  |  |  | 5'ACGTTGGATGGGCAGTTATGAGAAAGCACC3' | AGCACCAGCGGAGACG |
|  |  | rs3736228 | C/T (+) | NC_000011.10:g.68433827C>T | Missense | 5'ACGTTGGATGTCTTGGCAGAGCCTTGACG3' | gagcCTGTCAGGACCGCTCAGACGAGGC |
|  |  |  |  |  |  | 5'ACGTTGGATGACGGCGAGGCAGACTGTCA3' | gagcCTGTCAGGACCGCTCAGACGAGGT |
|  |  | rs556442 | A/G (+) | NC_000011.10:g.68425222A>G | Synonymous | 5'ACGTTGGATGTCTTCACCACCGGCCTCATC3' | TTGCCCAGTGTGTTGTCC |
|  |  |  |  |  |  | 5'ACGTTGGATGTCTCAATGCGCTTCAGGTCC3' | TTGCCCAGTGTGTTGTCT |
| EPDR1 | 7p14.1 | rs16879765 | C/T (+) | NC_000007.14:g.37949493C>T | Intron | 5'ACGTTGGATGATGTGAAGCCATTCTCTGCC3' | gggaTAAAAACTGTCTTGGGATAGGCA |
|  |  |  |  |  |  | 5'ACGTTGGATGACCATACTGTGTTCCACAGC3' | gggaTAAAAACTGTCTTGGGATAGGCG |
| LOC105375236 | 7p14.1 | rs1721400 | C/T (+) | NC_000007.14:g.38021183C>T | Intron | 5'ACGTTGGATGTCTCTCATTTCCCAACTCAC3' | aggaGAACTCCAGCCAATAACTCTA |
|  |  |  |  |  |  | 5'ACGTTGGATGGGCCCTGGATATCTATTTGG3' | aggaGAACTCCAGCCAATAACTCTG |
| SFRP4 | 7p14.1 | rs1052981 | A/G (-) | NC_000007.14:g.37906899A>G | 3' UTR | 5'ACGTTGGATGGGATTTTTGTGATGAAAGGG3' | AAGTCATTGTAAAAAAGACACATTATAA |
|  |  |  |  |  |  | 5'ACGTTGGATGAAACAGAGCTGAAGTCATTG3' | AAGTCATTGTAAAAAAGACACATTATAG |
|  |  | rs1376264 | G/A (-) | NC_000007.14:g.37918687G>A | Promoter | 5'ACGTTGGATGTCCTGATGCATCCTTCTCTG3' | GTGCCTGCCCCATTCA |
|  |  |  |  |  |  | 5'ACGTTGGATGTCCTAAAGCTTTGTGCCTGC3' | GTGCCTGCCCCATTCG |
|  |  | rs1802073 | T/G (-) | NC_000007.14:g.37907562T>G | Missense | 5'ACGTTGGATGGTAATCCCCCCAAACCAAAG3' | GTTTGGGAGCAGGAGG |
|  |  |  |  |  |  | 5'ACGTTGGATGTTGTTCTCTTCTGGGCACTC3' | GTTTGGGAGCAGGAGT |
|  |  | rs2084651 | C/G (-) | NC_000007.14:g.37917506C>G | Promoter | 5'ACGTTGGATGAGGGAGTTAGAAGGAAGAGG3' | cttttTGCTAGCATCTTCCAGCTTC |
|  |  |  |  |  |  | 5'ACGTTGGATGCTGCTCCAGGAAGTTAACAG3' | cttttTGCTAGCATCTTCCAGCTTG |
|  |  | rs2598116 | A/C (-) | NC_000007.14:g.37906746A>C | 3' UTR | 5'ACGTTGGATGGGCAGACAATGTCTGGATTC3' | AGAGGGTGTTGACAAGC |
|  |  |  |  |  |  | 5'ACGTTGGATGTTCTGGTGCTGCTTAAGAGG3' | AGAGGGTGTTGACAAGA |
|  |  | rs1802074 | C/T (-) | NC_000007.14:g.37907501C>T | Missense | 5'ACGTTGGATGGTAAGGAAGTCGGAAGTCTC3' | ggAGGAGTGCCCAGAAGAA |
|  |  |  |  |  |  | 5'ACGTTGGATGCATTAAAACTAGGAGTGCCC3' | ggAGGAGTGCCCAGAAGAG |

Chr, chromosome; forward (+) or reverse (-) reads behind variant alleles represent the orientation of genome. Gsk3β, glycogen synthase kinase 3 beta; LRP5, LDL receptor related protein 5; EPDR1, ependymin related 1; LOC105375236, uncharacterized; SFRP4, secreted frizzled related protein 4.
