# Supplementary material for: 17 variants interaction of Wnt/β-catenin pathway associated with development of osteonecrosis of femoral head in Chinese Han population
Source: Sci Rep. 2024 Mar 27;14:7301. doi: 10.1038/s41598-024-57929-8 (PMC10973331; doi:10.1038/s41598-024-57929-8)
Supplement: Supplementary file 1 — Supplementary Tables. [file 41598_2024_57929_MOESM1_ESM.zip › Supplementary Tables/Supplementary Table 2.docx]

**Supplementary Table 2. Association of the genotypes and allele frequencies of 17 variantsin Wnt/β-catenin pathway with ONFH risk.**

| **Gene** | **Variant** | **Group** | **Genotype (n + %)** | | | **MAF** | **HWE** | **P^a^** | **Co-dominants (11 vs. 12 vs. 22)** | **Dominants (12 + 22 vs. 11)** | **Recessives (22 vs. 11 + 12)** | **Allele 2 vs. 1** |
| --- | --- | --- | --- | --- | --- | --- | --- | --- | --- | --- | --- | --- |
|  |  |  | 11 | 12 | 22 |  |  |  | OR (95% CI) P^b^ | OR (95% CI) P^b^ | OR (95% CI) P^b^ | OR (95% CI) P^b^ |
| Gsk3β | rs2037547 (C/T) |  | CC | CT | TT |  |  |  |  |  |  |  |
|  |  | Control | 255 (85.3) | 44 (14.7) | 0 (0.0) | 0.074 | 0.385 | **0.059** | 0.701 (0.406-1.213) | 0.776 (0.576-1.046) | — | 0.863 (0.558-1.334) |
|  |  | ONFH | 230 (88.5) | 27 (10.4) | 3 (1.2) | 0.063 | 0.071 |  | 0.204 | **0.096** | — | 0.505 |
|  | rs334558 (G/A) |  | GG | GA | AA |  |  |  |  |  |  |  |
|  |  | Control | 85 (29.8) | 150 (52.6) | 50 (17.5) | 0.439 | 0.280 | **0.001** | 1.622 (1.189-2.213) | 1.377 (1.072-1.768) | 1.360 (1.061-1.743) | 1.259 (1.114-1.422) |
|  |  | ONFH | 45 (18.1) | 133 (53.4) | 71 (28.5) | 0.552 | 0.249 |  | **0.002** | **0.012** | **0.015** | **0.0002** |
|  | rs3732361 (A/G) |  | AA | AG | GG |  |  |  |  |  |  |  |
|  |  | Control | 110 (37.3) | 131 (44.4) | 54 (18.3) | 0.405 | 0.184 | 0.491 | 1.284 (0.967-1.705) | 1.229 (0.991-1.523) | 1.121 (0.867-1.448) | 1.091 (0.950-1.252) |
|  |  | ONFH | 85 (32.9) | 118 (45.7) | 55 (21.3) | 0.442 | 0.256 |  | **0.084** | **0.060** | 0.383 | 0.217 |
|  | rs3755557 (T/A) |  | TT | TA | AA |  |  |  |  |  |  |  |
|  |  | Control | 198 (67.6) | 85 (29.0) | 10 (3.4) | 0.179 | 0.842 | 0.724 | 0.745 (0.502-1.107) | 0.878 (0.702-1.097) | 0.606 (0.311-1.183) | 0.898 (0.691-1.167) |
|  |  | ONFH | 182 (70.5) | 69 (26.7) | 7 (2.7) | 0.161 | 0.819 |  | 0.145 | 0.252 | 0.142 | 0.420 |
|  | rs6438552 (G/A) |  | GG | GA | AA |  |  |  |  |  |  |  |
|  |  | Control | 109 (37.2) | 132 (45.1) | 52 (17.7) | 0.403 | 0.276 | 0.650 | 1.209 (0.910-1.606) | 1.163 (0.939-1.440) | 1.098 (0.847-1.423) | 1.070 (0.930-1.231) |
|  |  | ONFH | 87 (34.4) | 114 (45.1) | 52 (20.6) | 0.431 | 0.201 |  | 0.190 | 0.166 | 0.479 | 0.347 |
| LRP5 | rs2306862 (C/T) |  | CC | CT | TT |  |  |  |  |  |  |  |
|  |  | Control | 188 (62.7) | 97 (32.3) | 15 (5.0) | 0.212 | 0.604 | 0.860 | 1.203 (0.859-1.684) | 1.130 (0.919-1.390) | 1.079 (0.694-1.679) | 1.003 (0.800-1.258) |
|  |  | ONFH | 160 (61.8) | 88 (34.0) | 11 (4.2) | 0.212 | 1.000 |  | 0.282 | 0.246 | 0.734 | 0.978 |
|  | rs312778 (T/C) |  | TT | TC | CC |  |  |  |  |  |  |  |
|  |  | Control | 238 (80.1) | 58 (19.5) | 1 (0.3) | 0.101 | 0.333 | 0.346 | 0.539 (0.316-0.921) | 0.735 (0.562-0.961) | — | 0.803 (0.551-1.169) |
|  |  | ONFH | 217 (83.8) | 42 (16.2) | 0 (0.0) | 0.081 | 0.389 |  | **0.024** | **0.024** | — | 0.251 |
|  | rs3736228 (C/T) |  | CC | CT | TT |  |  |  |  |  |  |  |
|  |  | Control | 179 (60.1) | 103 (34.6) | 16 (5.4) | 0.227 | 0.869 | 0.569 | 1.182 (0.845-1.656) | 1.130 (0.921-1.385) | 1.004 (0.639-1.578) | 1.002 (0.806-1.245) |
|  |  | ONFH | 152 (58.5) | 98 (37.7) | 10 (3.8) | 0.227 | 0.290 |  | 0.329 | 0.242 | 0.986 | 0.987 |
|  | rs556442 (A/G) |  | AA | AG | GG |  |  |  |  |  |  |  |
|  |  | Control | 146 (49.0) | 118 (39.6) | 34 (11.4) | 0.312 | 0.179 | 0.137 | 1.005 (0.738-1.368) | 1.066 (0.871-1.305) | 0.835 (0.584-1.192) | 0.917 (0.765-1.100) |
|  |  | ONFH | 126 (49.4) | 112 (43.9) | 17 (6.7) | 0.286 | 0.284 |  | 0.976 | 0.536 | 0.321 | 0.351 |
| EPDR1 | rs16879765 (C/T) |  | CC | CT | TT |  |  |  |  |  |  |  |
|  |  | Control | 244 (81.9) | 51 (17.1) | 3 (1.0) | 0.096 | 0.740 | 0.985 | 1.075 (0.661-1.749) | 1.045 (0.803-1.360) | 0.975 (0.346-2.747) | 1.009 (0.703-1.448) |
|  |  | ONFH | 212 (81.9) | 44 (17.0) | 3 (1.2) | 0.097 | 0.716 |  | 0.770 | 0.742 | 0.962 | 0.960 |
| LOC105375236 | rs1721400 (C/T) |  | CC | CT | TT |  |  |  |  |  |  |  |
|  |  | Control | 191 (64.3) | 97 (32.7) | 9 (3.0) | 0.194 | 0.576 | 0.117 | 0.785 (0.541-1.140) | 0.854 (0.687-1.061) | 0.961 (0.546-1.692) | 0.798 (0.615-1.034) |
|  |  | ONFH | 187 (72.2) | 64 (24.7) | 8 (3.1) | 0.154 | 0.348 |  | 0.204 | 0.154 | 0.892 | **0.087** |
| SFRP4 | rs1052981 (A/G) |  | AA | AG | GG |  |  |  |  |  |  |  |
|  |  | Control | 213 (71.7) | 73 (24.6) | 11 (3.7) | 0.160 | 0.134 | 0.480 | 1.198 (0.834-1.720) | 1.028 (0.825-1.282) | 1.708 (1.041-2.805) | 1.103 (0.848-1.435) |
|  |  | ONFH | 180 (70.6) | 60 (23.5) | 15 (5.9) | 0.176 | 0.004 |  | 0.329 | 0.804 | **0.034** | 0.463 |
|  | rs1376264 (G/A) |  | GG | GA | AA |  |  |  |  |  |  |  |
|  |  | Control | 196 (66.9) | 82 (28.0) | 15 (5.1) | 0.191 | 0.128 | 0.891 | 1.219 (0.857-1.735) | 1.109 (0.891-1.381) | 1.245 (0.787-1.969) | 1.009 (0.791-1.288) |
|  |  | ONFH | 171 (67.3) | 68 (26.8) | 15 (5.9) | 0.193 | 0.028 |  | 0.271 | 0.355 | 0.348 | 0.940 |
|  | rs1802073 (T/G) |  | TT | TG | GG |  |  |  |  |  |  |  |
|  |  | Control | 69 (23.2) | 158 (53.2) | 70 (23.6) | 0.502 | 0.298 | 0.161 | 0.960 (0.702-1.312) | 1.077 (0.843-1.377) | 0.872 (0.674-1.129) | 0.953 (0.844-1.076) |
|  |  | ONFH | 55 (22.0) | 151 (60.4) | 44 (17.6) | 0.478 | 0.001 |  | 0.796 | 0.553 | 0.299 | 0.435 |
|  | rs2084651 (C/G) |  | CC | CG | GG |  |  |  |  |  |  |  |
|  |  | Control | 97 (32.9) | 146 (49.5) | 52 (17.6) | 0.424 | 0.905 | 0.925 | 1.131 (0.845-1.512) | 1.056 (0.854-1.306) | 1.131 (0.865-1.479) | 0.987 (0.859-1.134) |
|  |  | ONFH | 88 (34.2) | 123 (47.9) | 46 (17.9) | 0.418 | 0.798 |  | 0.408 | 0.614 | 0.369 | 0.855 |
|  | rs2598116 (A/C) |  | AA | AC | CC |  |  |  |  |  |  |  |
|  |  | Control | 174 (58.0) | 107 (35.7) | 19 (6.3) | 0.242 | 0.638 | 0.749 | 0.762 (0.545-1.066) | 0.851 (0.692-1.046) | 0.821 (0.531-1.269) | 0.923 (0.745-1.143) |
|  |  | ONFH | 159 (61.2) | 86 (33.1) | 15 (5.8) | 0.223 | 0.474 |  | 0.113 | 0.125 | 0.374 | 0.463 |
|  | rs1802074 (C/T) |  | CC | CT | TT |  |  |  |  |  |  |  |
|  |  | Control | 170 (57.6) | 114 (38.6) | 11 (3.7) | 0.231 | 0.142 | 0.772 | 1.052 (0.742-1.492) | 1.015 (0.828-1.245) | 1.127 (0.682-1.860) | 0.995 (0.801-1.236) |
|  |  | ONFH | 150 (58.8) | 93 (36.5) | 12 (4.7) | 0.229 | 0.724 |  | 0.776 | 0.886 | 0.641 | 0.966 |

11, major allele homozygote; 12, heterozygote; 22, minor allele homozygote. MAF, minor allele frequency; HWE, Hardy–Weinberg equilibrium.

**^a^**χ^2^ test (or Fisher exact test); **^b^**Logistic regression analyses.
