# Supplementary material for: 17 variants interaction of Wnt/β-catenin pathway associated with development of osteonecrosis of femoral head in Chinese Han population
Source: Sci Rep. 2024 Mar 27;14:7301. doi: 10.1038/s41598-024-57929-8 (PMC10973331; doi:10.1038/s41598-024-57929-8)
Supplement: Supplementary file 1 — Supplementary Tables. [file 41598_2024_57929_MOESM1_ESM.zip › Supplementary Tables/Supplementary Table 3.docx]

**Supplementary Table 3. Association of the genotypes of 17 variants in Wnt/β-catenin pathway with the clinical traits of ONFH.**

| Gene | Variant | genotype | Gender n (%)a | | Age at onset (year)b | Etiological classification n (%)a | | | Hip lesions n (%)a | | Clinical stages n (%)a | | |
| --- | --- | --- | --- | --- | --- | --- | --- | --- | --- | --- | --- | --- | --- |
|  |  |  | Male | Female |  | Alc | Ster | Idio | Unilateral | Bilateral | Stage Ⅱ | Stage Ⅲ | Stage Ⅳ |
| Gsk3β | rs2037547(C/T) | CC | 273 (86.7) | 212 (86.9) | 48.88±12.95 | 102 (86.4) | 48 (88.9) | 80 (90.9) | 113 (92.6) | 117 (84.8) | 18 (90.0) | 75 (89.3) | 137 (87.8) |
|  |  | CT | 39 (12.4) | 32 (13.1) | 45.65±11.09 | 13 (11.0) | 6 (11.1) | 8 (9.1) | 7 (5.7) | 20 (14.5) | 2 (10.0) | 8 (9.5) | 17 (10.9) |
|  |  | TT | 3 (1.0) | 0 (0.0) | 55.00±8.54 | 3 (2.5) | 0 (0.0) | 0 (0.0) | 2 (1.6) | 1 (0.7) | 0 (0.0) | 1 (1.2) | 2 (1.3) |
|  |  | P | 0.304 |  | 0.344 | 0.612 |  |  | ***0.058*** |  | 0.982 |  |  |
|  | rs334558(G/A) | GG | 68 (22.8) | 62 (26.3) | 47.59±11.74 | 23 (20.9) | 8 (15.4) | 14 (16.1) | 24 (20.0) | 21 (16.3) | 1 (5.9) | 16 (20.0) | 28 (18.4) |
|  |  | GA | 158 (53.0) | 125 (53.0) | 48.15±13.39 | 57 (51.8) | 27 (51.9) | 49 (56.3) | 60 (50.0) | 73 (56.6) | 13 (76.5) | 46 (57.5) | 74 (48.7) |
|  |  | AA | 72 (24.2) | 49 (20.8) | 49.70±12.27 | 30 (27.3) | 17 (32.7) | 24 (27.6) | 36 (30.0) | 35 (27.1) | 3 (17.6) | 18 (22.5) | 50 (32.9) |
|  |  | P | 0.518 |  | 0.648 | 0.826 |  |  | 0.560 |  | 0.142 |  |  |
|  | rs3732361(A/G) | AA | 112 (36.0) | 83 (34.3) | 46.50±10.91 | 43 (36.8) | 17 (32.1) | 25 (28.4) | 39 (32.2) | 46 (33.6) | 4 (20.0) | 30 (35.7) | 51 (33.1) |
|  |  | AG | 136 (43.7) | 113 (46.7) | 50.15±13.45 | 49 (41.9) | 24 (45.3) | 45 (51.1) | 57 (47.1) | 61 (44.5) | 11 (55.0) | 38 (45.2) | 69 (44.8) |
|  |  | GG | 63 (20.3) | 46 (19.0) | 48.41±13.45 | 25 (21.4) | 12 (22.6) | 18 (20.5) | 25 (20.7) | 30 (21.9) | 5 (25.0) | 16 (19.0) | 34 (22.1) |
|  |  | P | 0.784 |  | 0.143 | 0.718 |  |  | 0.916 |  | 0.737 |  |  |
|  | rs3755557(T/A) | TT | 226 (72.7) | 154 (64.2) | 50.43±12.98 | 91 (77.8) | 33 (62.3) | 58 (65.9) | 81 (67.5) | 101 (73.2) | 15 (78.9) | 57 (68.7) | 110 (70.5) |
|  |  | TA | 75 (24.1) | 79 (32.9) | 45.04±11.39 | 22 (18.8) | 17 (32.1) | 30 (34.1) | 34 (28.3) | 35 (25.4) | 4 (21.1) | 21 (25.3) | 44 (28.2) |
|  |  | AA | 10 (3.2) | 7 (2.9) | 38.21±8.62 | 4 (3.4) | 3 (5.7) | 0 (0.0) | 5 (4.2) | 2 (1.4) | 0 (0.0) | 5 (6.0) | 2 (1.3) |
|  |  | P | ***0.074*** |  | **0.001** | **0.026** |  |  | 0.324 |  | 0.310 |  |  |
|  | rs6438552(G/A) | GG | 112 (36.5) | 84 (35.1) | 46.79±11.22 | 43 (38.4) | 19 (35.2) | 25 (28.7) | 40 (34.2) | 47 (34.6) | 5 (26.3) | 29 (36.3) | 53 (34.4) |
|  |  | GA | 134 (43.6) | 112 (46.9) | 50.13±13.60 | 45 (40.2) | 23 (42.6) | 46 (52.9) | 54 (46.2) | 60 (44.1) | 10 (52.6) | 37 (46.3) | 67 (43.5) |
|  |  | AA | 61 (19.9) | 43 (18.0) | 48.43±13.28 | 24 (21.4) | 12 (22.2) | 16 (18.4) | 23 (19.7) | 29 (21.3) | 4 (21.1) | 14 (17.5) | 34 (22.1) |
|  |  | P | 0.732 |  | 0.200 | 0.478 |  |  | 0.930 |  | 0.856 |  |  |
| LRP5 | rs2306862(C/T) | CC | 205 (65.3) | 143 (58.4) | 48.34±12.84 | 77 (65.8) | 30 (55.6) | 53 (60.2) | 73 (60.3) | 87 (63.0) | 13 (65.0) | 54 (64.3) | 93 (60.0) |
|  |  | CT | 95 (30.3) | 90 (36.7) | 48.40±12.79 | 35 (29.9) | 23 (42.6) | 30 (34.1) | 43 (35.5) | 45 (32.6) | 5 (25.0) | 28 (33.3) | 55 (35.5) |
|  |  | TT | 14 (4.5) | 12 (4.9) | 55.89±10.82 | 5 (4.3) | 1 (1.9) | 5 (5.7) | 5 (4.1) | 6 (4.3) | 2 (10.0) | 2 (2.4) | 7 (4.5) |
|  |  | P | 0.239 |  | 0.224 | 0.465 |  |  | 0.884 |  | 0.505 |  |  |
|  | rs312778(T/C) | TT | 246 (78.8) | 209 (85.7) | 48.85±12.57 | 94 (80.3) | 47 (87.0) | 76 (86.4) | 104 (85.2) | 113 (82.5) | 18 (90.0) | 71 (85.5) | 128 (82.1) |
|  |  | TC | 65 (20.8) | 35 (14.3) | 47.17±13.75 | 23 (19.7) | 7 (13.0) | 12 (13.6) | 18 (14.8) | 24 (17.5) | 2 (10.0) | 12 (14.5) | 28 (17.9) |
|  |  | CC | 1 (0.3) | 0 (0.0) | — | — | — | — | — | — | — | — | — |
|  |  | P | ***0.058*** |  | 0.453 | 0.392 |  |  | 0.547 |  | 0.576 |  |  |
|  | rs3736228(C/T) | CC | 196 (62.2) | 135 (55.6) | 48.04±12.62 | 72 (61.0) | 31 (57.4) | 49 (55.7) | 70 (57.4) | 82 (59.4) | 11 (55.0) | 50 (59.5) | 91 (58.3) |
|  |  | CT | 106 (33.7) | 95 (39.1) | 48.65±13.09 | 41 (34.7) | 22 (40.7) | 35 (39.8) | 48 (39.3) | 50 (36.2) | 7 (35.0) | 32 (38.1) | 59 (37.8) |
|  |  | TT | 13 (4.1) | 13 (5.3) | 58.88±6.49 | 5 (4.2) | 1 (1.9) | 4 (4.5) | 4 (3.3) | 6 (4.3) | 2 (10.0) | 2 (2.4) | 6 (3.8) |
|  |  | P | 0.273 |  | ***0.065*** | 0.830 |  |  | 0.817 |  | 0.611 |  |  |
|  | rs556442(A/G) | AA | 161 (51.9) | 111 (45.7) | 47.70±13.12 | 60 (52.2) | 25 (47.2) | 41 (47.1) | 54 (46.2) | 72 (52.2) | 9 (45.0) | 39 (48.8) | 78 (50.3) |
|  |  | AG | 122 (39.4) | 108 (44.4) | 48.86±12.33 | 44 (38.3) | 26 (49.1) | 42 (48.3) | 56 (47.9) | 56 (40.6) | 9 (45.0) | 37 (46.3) | 66 (42.6) |
|  |  | GG | 27 (8.7) | 24 (9.9) | 49.80±10.91 | 11 (9.6) | 2 (3.8) | 4 (4.6) | 7 (6.0) | 10 (7.2) | 2 (10.0) | 4 (5.0) | 11 (7.1) |
|  |  | P | 0.344 |  | 0.713 | 0.339 |  |  | 0.501 |  | 0.911 |  |  |
| EPDR1 | rs16879765(C/T) | CC | 250 (79.9) | 206 (84.4) | 48.59±11.86 | 97 (82.9) | 46 (85.2) | 69 (78.4) | 99 (81.8) | 113 (81.9) | 18 (90.0) | 72 (86.7) | 122 (78.2) |
|  |  | CT | 58 (18.5) | 37 (15.2) | 48.65±16.05 | 17 (14.5) | 8 (14.8) | 19 (21.6) | 21 (17.4) | 23 (16.7) | 2 (10.0) | 10 (12.0) | 32 (20.5) |
|  |  | TT | 5 (1.6) | 1 (0.4) | 46.33±23.46 | 3 (2.6) | 0 (0.0) | 0 (0.0) | 1 (0.8) | 2 (1.4) | 0 (0.0) | 1 (1.2) | 2 (1.3) |
|  |  | P | 0.217 |  | 0.988 | 0.347 |  |  | 0.890 |  | 0.425 |  |  |
| LOC105375236 | rs1721400(C/T) | CC | 215 (68.9) | 163 (66.8) | 48.45±12.82 | 88 (75.2) | 39 (72.2) | 60 (68.2) | 89 (73.0) | 98 (71.5) | 16 (80.0) | 60 (71.4) | 111 (71.6) |
|  |  | CT | 89 (28.5) | 72 (29.5) | 47.91±12.45 | 26 (22.2) | 14 (25.9) | 24 (27.3) | 29 (23.8) | 35 (25.5) | 4 (20.0) | 20 (23.8) | 40 (25.8) |
|  |  | TT | 8 (2.6) | 9 (3.7) | 58.25±12.07 | 3 (2.6) | 1 (1.9) | 4 (4.5) | 4 (3.3) | 4 (2.9) | 0 (0.0) | 4 (4.8) | 4 (2.6) |
|  |  | P | 0.704 |  | ***0.093*** | 0.758 |  |  | 0.938 |  | 0.852 |  |  |
| SFRP4 | rs1052981(A/G) | AA | 220 (71.2) | 173 (71.2) | 49.07±12.63 | 85 (73.3) | 36 (67.9) | 59 (68.6) | 82 (67.8) | 98 (73.1) | 12 (60.0) | 61 (73.5) | 107 (70.4) |
|  |  | AG | 73 (23.6) | 60 (24.7) | 47.80±11.66 | 26 (22.4) | 12 (22.6) | 22 (25.6) | 29 (24.0) | 31 (23.1) | 6 (30.0) | 16 (19.3) | 38 (25) |
|  |  | GG | 16 (5.2) | 10 (4.1) | 51.18±17.07 | 5 (4.3) | 5 (9.4) | 5 (5.8) | 10 (8.3) | 5 (3.7) | 2 (10.0) | 6 (7.2) | 7 (4.6) |
|  |  | P | 0.823 |  | 0.641 | 0.722 |  |  | 0.287 |  | 0.591 |  |  |
|  | rs1376264(G/A) | GG | 204 (66.9) | 163 (67.4) | 48.57±12.39 | 77 (68.1) | 36 (66.7) | 58 (66.7) | 78 (65.5) | 93 (68.9) | 11 (55.0) | 56 (69.1) | 104 (68.0) |
|  |  | GA | 82 (26.9) | 68 (28.1) | 49.02±13.59 | 30 (26.5) | 14 (25.9) | 24 (27.6) | 33 (27.7) | 35 (25.9) | 7 (35.0) | 22 (27.2) | 39 (25.5) |
|  |  | AA | 19 (6.2) | 11 (4.5) | 47.97±15.53 | 6 (5.3) | 4 (7.4) | 5 (5.7) | 8 (6.7) | 7 (5.2) | 2 (10.0) | 3 (3.7) | 10 (6.5) |
|  |  | P | 0.679 |  | 0.953 | 0.987 |  |  | 0.804 |  | 0.655 |  |  |
|  | rs1802073(T/G) | TT | 67 (21.6) | 57 (24.1) | 48.21±12.93 | 25 (21.6) | 9 (18.4) | 21 (24.7) | 21 (17.9) | 34 (25.6) | 4 (21.1) | 16 (19.8) | 35 (23.3) |
|  |  | TG | 191 (61.6) | 118 (49.8) | 48.67±12.80 | 69 (59.5) | 32 (65.3) | 50 (58.8) | 74 (63.2) | 77 (57.9) | 12 (63.2) | 47 (58.0) | 92 (61.3) |
|  |  | GG | 52 (16.8) | 62 (26.2) | 49.34±12.99 | 22 (19.0) | 8 (16.3) | 14 (16.5) | 22 (18.8) | 22 (16.5) | 3 (15.8) | 18 (22.2) | 23 (15.3) |
|  |  | P | **0.009** |  | 0.916 | 0.900 |  |  | 0.347 |  | 0.756 |  |  |
|  | rs2084651(C/G) | CC | 102 (32.9) | 83 (34.3) | 49.17±11.48 | 37 (31.6) | 21 (39.6) | 30 (34.5) | 38 (31.4) | 50 (36.8) | 5 (25.0) | 27 (32.5) | 56 (36.4) |
|  |  | CG | 160 (51.6) | 109 (45.0) | 48.54±13.07 | 59 (50.4) | 21 (39.6) | 43 (49.4) | 61 (50.4) | 62 (45.6) | 10 (50.0) | 40 (48.2) | 73 (47.4) |
|  |  | GG | 48 (15.5) | 50 (20.7) | 48.56±14.33 | 21 (17.9) | 11 (20.8) | 14 (16.1) | 22 (18.2) | 24 (17.6) | 5 (25.0) | 16 (19.3) | 25 (16.2) |
|  |  | P | 0.189 |  | 0.939 | 0.728 |  |  | 0.651 |  | 0.792 |  |  |
|  | rs2598116(A/C) | AA | 181 (57.5) | 152 (62.0) | 49.49±12.87 | 72 (61.0) | 35 (64.8) | 52 (59.1) | 77 (63.1) | 82 (59.4) | 13 (65.0) | 56 (66.7) | 90 (57.7) |
|  |  | AC | 119 (37.8) | 74 (30.2) | 47.54±12.29 | 40 (33.9) | 15 (27.8) | 31 (35.2) | 43 (35.2) | 43 (31.2) | 5 (25.0) | 22 (26.2) | 59 (37.8) |
|  |  | CC | 15 (4.8) | 19 (7.8) | 45.87±14.07 | 6 (5.1) | 4 (7.4) | 5 (5.7) | 2 (1.6) | 13 (9.4) | 2 (10.0) | 6 (7.1) | 7 (4.5) |
|  |  | P | ***0.090*** |  | 0.380 | 0.889 |  |  | **0.026** |  | 0.312 |  |  |
|  | rs1802074(C/T) | CC | 176 (56.6) | 144 (60.3) | 48.38±13.07 | 68 (58.1) | 32 (60.4) | 50 (58.8) | 74 (61.7) | 76 (56.3) | 13 (65.0) | 47 (58.0) | 90 (58.4) |
|  |  | CT | 122 (39.2) | 85 (35.6) | 48.88±12.03 | 45 (38.5) | 19 (35.8) | 29 (34.1) | 42 (35.0) | 51 (37.8) | 5 (25.0) | 31 (38.3) | 57 (37.0) |
|  |  | TT | 13 (4.2) | 10 (4.2) | 48.83±16.34 | 4 (3.4) | 2 (3.8) | 6 (7.1) | 4 (3.3) | 8 (5.9) | 2 (10.0) | 3 (3.7) | 7 (4.5) |
|  |  | P | 0.673 |  | 0.959 | 0.772 |  |  | 0.508 |  | 0.610 |  |  |

Alc: Alcohol-induced; Ster: steroid- induced; Idio: idiopathic. a, χ2 test (or Fisher exact test); b, one-way ANOVA (or Tukey test).
