# Supplementary material for: 17 variants interaction of Wnt/β-catenin pathway associated with development of osteonecrosis of femoral head in Chinese Han population
Source: Sci Rep. 2024 Mar 27;14:7301. doi: 10.1038/s41598-024-57929-8 (PMC10973331; doi:10.1038/s41598-024-57929-8)
Supplement: Supplementary file 1 — Supplementary Tables. [file 41598_2024_57929_MOESM1_ESM.zip › Supplementary Tables/Supplementary Table 4.docx]

**Supplementary Table 4. Association of paired gene-gene interactions of 17 variants in Wnt/β-catenin pathway with ONFH risk.**

|  |  | **Gsk3β** | | | | | **LRP5** | | | | **EPDR1** | **LOC105375236** | **SFRP4** | | | | | |
| --- | --- | --- | --- | --- | --- | --- | --- | --- | --- | --- | --- | --- | --- | --- | --- | --- | --- | --- |
|  |  | rs2037547 | rs334558 | rs3732361 | rs3755557 | rs6438552 | rs2306862 | rs312778 | rs3736228 | rs556442 | rs16879765 | rs1721400 | rs1052981 | rs1376264 | rs1802073 | rs2084651 | rs2598116 | rs1802074 |
| Gsk3β | rs2037547 | — | 0.759 | 0.778 | 0.676 | 0.641 | 1.139 | 0.371 | 1.258 | 0.960 | 0.318 | 0.673 | 0.684 | 0.776 | 0.801 | 0.842 | 0.747 | 0.593 |
|  |  | — | 0.398-1.447 | 0.296-2.046 | 0.281-1.627 | 0.231-1.781 | 0.509-2.548 | 0.106-1.305 | 0.533-2.972 | 0.471-1.956 | 0.045-2.241 | 0.309-1.466 | 0.316-1.481 | 0.349-1.728 | 0.479-1.338 | 0.510-1.391 | 0.390-1.431 | 0.271-1.297 |
|  |  | — | 0.402 | 0.611 | 0.382 | 0.394 | 0.752 | 0.122 | 0.600 | 0.910 | 0.250 | 0.318 | 0.336 | 0.535 | 0.397 | 0.502 | 0.379 | 0.190 |
|  | rs334558 | 0.759 | — | 1.127 | 1.307 | 1.096 | 1.362 | 0.896 | 1.343 | 1.113 | 1.249 | 0.896 | 1.172 | 1.218 | 1.093 | 1.158 | 1.066 | 1.387 |
|  |  | 0.398-1.447 | — | 0.961-1.321 | 0.856-1.993 | 0.935-1.284 | 1.036-1.789 | 0.590-1.363 | 1.031-1.751 | 0.893-1.387 | 0.831-1.877 | 0.657-1.222 | 0.866-1.585 | 0.911-1.629 | 0.895-1.334 | 0.950-1.411 | 0.811-1.401 | 1.057-1.821 |
|  |  | 0.402 | — | 0.142 | 0.215 | 0.258 | **0.027** | 0.609 | **0.029** | 0.342 | 0.285 | 0.489 | 0.303 | 0.183 | 0.382 | 0.147 | 0.648 | **0.018** |
|  | rs3732361 | 0.778 | 1.127 | — | 1.137 | 1.101 | 1.261 | 0.902 | 1.291 | 1.125 | 1.411 | 0.983 | 1.271 | 1.296 | 1.066 | 1.169 | 1.080 | 1.121 |
|  |  | 0.296-2.046 | 0.961-1.321 | — | 0.626-2.067 | 0.953-1.273 | 0.962-1.653 | 0.551-1.474 | 0.994-1.677 | 0.903-1.402 | 0.884-2.253 | 0.706-1.369 | 0.922-1.751 | 0.944-1.778 | 0.871-1.305 | 0.952-1.436 | 0.820-1.422 | 0.843-1.492 |
|  |  | 0.611 | 0.142 | — | 0.673 | 0.190 | 0.093 | 0.680 | 0.055 | 0.295 | 0.149 | 0.921 | 0.143 | 0.109 | 0.536 | 0.136 | 0.584 | 0.432 |
|  | rs3755557 | 0.676 | 1.307 | 1.137 | — | 1.167 | 0.825 | 0.617 | 0.797 | 0.720 | 0.728 | 0.687 | 0.794 | 0.902 | 0.855 | 0.922 | 0.611 | 0.965 |
|  |  | 0.281-1.627 | 0.856-1.993 | 0.626-2.067 | — | 0.639-2.131 | 0.503-1.353 | 0.270-1.409 | 0.490-1.295 | 0.471-1.100 | 0.353-1.504 | 0.401-1.177 | 0.475-1.330 | 0.548-1.486 | 0.629-1.163 | 0.664-1.280 | 0.382-0.976 | 0.573-1.624 |
|  |  | 0.382 | 0.215 | 0.673 | — | 0.615 | 0.446 | 0.252 | 0.359 | 0.129 | 0.392 | 0.172 | 0.381 | 0.686 | 0.318 | 0.626 | **0.039** | 0.893 |
|  | rs6438552 | 0.641 | 1.096 | 1.101 | 1.167 | — | 1.236 | 0.882 | 1.268 | 1.108 | 1.452 | 0.977 | 1.266 | 1.317 | 1.041 | 1.148 | 1.029 | 1.085 |
|  |  | 0.231-1.781 | 0.935-1.284 | 0.953-1.273 | 0.639-2.131 | — | 0.937-1.630 | 0.541-1.438 | 0.972-1.655 | 0.886-1.387 | 0.907-2.325 | 0.693-1.376 | 0.917-1.748 | 0.957-1.814 | 0.850-1.274 | 0.932-1.413 | 0.773-1.370 | 0.814-1.445 |
|  |  | 0.394 | 0.258 | 0.190 | 0.615 | — | 0.133 | 0.614 | **0.080** | 0.367 | 0.121 | 0.892 | 0.152 | **0.091** | 0.701 | 0.194 | 0.844 | 0.580 |
| LRP5 | rs2306862 | 1.139 | 1.362 | 1.261 | 0.825 | 1.236 | — | 0.772 | 1.086 | 1.057 | 0.831 | 0.993 | 1.543 | 1.298 | 1.038 | 1.117 | 0.933 | 1.178 |
|  |  | 0.509-2.548 | 1.036-1.789 | 0.962-1.653 | 0.503-1.353 | 0.937-1.630 | — | 0.317-1.884 | 0.874-1.349 | 0.853-1.309 | 0.425-1.622 | 0.626-1.575 | 0.920-2.587 | 0.823-2.046 | 0.789-1.364 | 0.840-1.486 | 0.628-1.387 | 0.812-1.709 |
|  |  | 0.752 | **0.027** | **0.093** | 0.446 | 0.133 | — | 0.570 | 0.456 | 0.613 | 0.586 | 0.976 | 0.100 | 0.262 | 0.792 | 0.445 | 0.732 | 0.388 |
|  | rs312778 | 0.371 | 0.896 | 0.902 | 0.617 | 0.882 | 0.772 | — | 0.868 | 0.619 | 0.977 | 0.390 | 0.665 | 0.724 | 0.617 | 0.595 | 0.851 | 0.331 |
|  |  | 0.106-1.305 | 0.590-1.363 | 0.551-1.474 | 0.270-1.409 | 0.541-1.438 | 0.317-1.884 | — | 0.415-1.815 | 0.372-1.031 | 0.434-2.199 | 0.175-0.868 | 0.304-1.455 | 0.362-1.450 | 0.386-0.987 | 0.348-1.015 | 0.464-1.561 | 0.157-0.696 |
|  |  | 0.122 | 0.609 | 0.680 | 0.252 | 0.614 | 0.570 | — | 0.706 | **0.065** | 0.955 | **0.021** | 0.307 | 0.362 | **0.044** | **0.057** | 0.602 | **0.004** |
|  | rs3736228 | 1.258 | 1.343 | 1.291 | 0.797 | 1.268 | 1.086 | 0.868 | — | 1.034 | 1.026 | 1.129 | 1.486 | 1.270 | 1.003 | 1.114 | 0.974 | 1.059 |
|  |  | 0.533-2.972 | 1.031-1.751 | 0.994-1.677 | 0.490-1.295 | 0.972-1.655 | 0.874-1.349 | 0.415-1.815 | — | 0.835-1.280 | 0.559-1.881 | 0.721-1.768 | 0.947-2.330 | 0.833-1.938 | 0.768-1.309 | 0.845-1.467 | 0.655-1.448 | 0.734-1.528 |
|  |  | 0.600 | **0.029** | **0.055** | 0.359 | **0.080** | 0.456 | 0.706 | — | 0.759 | 0.934 | 0.596 | **0.085** | 0.267 | 0.985 | 0.444 | 0.897 | 0.760 |
|  | rs556442 | 0.960 | 1.113 | 1.125 | 0.720 | 1.108 | 1.057 | 0.619 | 1.034 | — | 0.877 | 0.925 | 1.078 | 1.125 | 0.932 | 1.015 | 1.022 | 0.991 |
|  |  | 0.471-1.956 | 0.893-1.387 | 0.903-1.402 | 0.471-1.100 | 0.886-1.387 | 0.853-1.309 | 0.372-1.031 | 0.835-1.280 | — | 0.535-1.437 | 0.621-1.377 | 0.745-1.561 | 0.787-1.610 | 0.735-1.181 | 0.794-1.298 | 0.725-1.442 | 0.714-1.374 |
|  |  | 0.910 | 0.342 | 0.295 | 0.129 | 0.367 | 0.613 | **0.065** | 0.759 | — | 0.601 | 0.699 | 0.691 | 0.518 | 0.558 | 0.904 | 0.900 | 0.956 |
| EPDR1 | rs16879765 | 0.318 | 1.249 | 1.411 | 0.728 | 1.452 | 0.831 | 0.977 | 1.026 | 0.877 | — | 0.899 | 1.032 | 0.917 | 1.019 | 1.012 | 0.440 | 0.942 |
|  |  | 0.045-2.241 | 0.831-1.877 | 0.884-2.253 | 0.353-1.504 | 0.907-2.325 | 0.425-1.622 | 0.434-2.199 | 0.559-1.881 | 0.535-1.437 | — | 0.391-2.067 | 0.656-1.624 | 0.571-1.475 | 0.737-1.409 | 0.733-1.398 | 0.156-1.241 | 0.442-2.009 |
|  |  | 0.250 | 0.285 | 0.149 | 0.392 | 0.121 | 0.586 | 0.955 | 0.934 | 0.601 | — | 0.803 | 0.891 | 0.722 | 0.908 | 0.942 | 0.121 | 0.878 |
| LOC105375236 | rs1721400 | 0.673 | 0.896 | 0.983 | 0.687 | 0.977 | 0.993 | 0.390 | 1.129 | 0.925 | 0.899 | — | 0.760 | 0.739 | 0.872 | 0.873 | 0.733 | 0.743 |
|  |  | 0.309-1.466 | 0.657-1.222 | 0.706-1.369 | 0.401-1.177 | 0.693-1.376 | 0.626-1.575 | 0.175-0.868 | 0.721-1.768 | 0.621-1.377 | 0.391-2.067 | — | 0.401-1.439 | 0.412-1.325 | 0.619-1.227 | 0.605-1.260 | 0.513-1.048 | 0.439-1.258 |
|  |  | 0.318 | 0.489 | 0.921 | 0.172 | 0.892 | 0.976 | **0.021** | 0.596 | 0.699 | 0.803 | — | 0.399 | 0.310 | 0.430 | 0.469 | **0.089** | 0.269 |
| SFRP4 | rs1052981 | 0.684 | 1.172 | 1.271 | 0.794 | 1.266 | 1.543 | 0.665 | 1.486 | 1.078 | 1.032 | 0.760 | — | 0.975 | 1.094 | 1.074 | 0.648 | 0.745 |
|  |  | 0.316-1.481 | 0.866-1.585 | 0.922-1.751 | 0.475-1.330 | 0.917-1.748 | 0.920-2.587 | 0.304-1.455 | 0.947-2.330 | 0.745-1.561 | 0.656-1.624 | 0.401-1.439 | — | 0.736-1.292 | 0.822-1.457 | 0.838-1.375 | 0.311-1.350 | 0.412-1.346 |
|  |  | 0.336 | 0.303 | 0.143 | 0.381 | 0.152 | 0.100 | 0.307 | **0.085** | 0.691 | 0.891 | 0.399 | — | 0.863 | 0.538 | 0.573 | 0.247 | 0.330 |
|  | rs1376264 | 0.776 | 1.218 | 1.296 | 0.902 | 1.317 | 1.298 | 0.724 | 1.270 | 1.125 | 0.917 | 0.739 | 0.975 | — | 1.135 | 1.142 | 0.829 | 1.187 |
|  |  | 0.349-1.728 | 0.911-1.629 | 0.944-1.778 | 0.548-1.486 | 0.957-1.814 | 0.823-2.046 | 0.362-1.450 | 0.833-1.938 | 0.787-1.610 | 0.571-1.475 | 0.412-1.325 | 0.736-1.292 | — | 0.859-1.501 | 0.931-1.401 | 0.416-1.649 | 0.846-1.666 |
|  |  | 0.535 | 0.183 | 0.109 | 0.686 | **0.091** | 0.262 | 0.362 | 0.267 | 0.518 | 0.722 | 0.310 | 0.863 | — | 0.373 | 0.203 | 0.592 | 0.320 |
|  | rs1802073 | 0.801 | 1.093 | 1.066 | 0.855 | 1.041 | 1.038 | 0.617 | 1.003 | 0.932 | 1.019 | 0.872 | 1.094 | 1.135 | — | 1.002 | 0.786 | 1.227 |
|  |  | 0.479-1.338 | 0.895-1.334 | 0.871-1.305 | 0.629-1.163 | 0.850-1.274 | 0.789-1.364 | 0.386-0.987 | 0.768-1.309 | 0.735-1.181 | 0.737-1.409 | 0.619-1.227 | 0.822-1.457 | 0.859-1.501 | — | 0.840-1.194 | 0.489-1.263 | 0.794-1.896 |
|  |  | 0.397 | 0.382 | 0.536 | 0.318 | 0.701 | 0.792 | **0.044** | 0.985 | 0.558 | 0.908 | 0.430 | 0.538 | 0.373 | — | 0.984 | 0.320 | 0.356 |
|  | rs2084651 | 0.842 | 1.158 | 1.169 | 0.922 | 1.148 | 1.117 | 0.595 | 1.114 | 1.015 | 1.012 | 0.873 | 1.074 | 1.142 | 1.002 | — | 0.787 | 1.098 |
|  |  | 0.510-1.391 | 0.950-1.411 | 0.952-1.436 | 0.664-1.280 | 0.932-1.413 | 0.840-1.486 | 0.348-1.015 | 0.845-1.467 | 0.794-1.298 | 0.733-1.398 | 0.605-1.260 | 0.838-1.375 | 0.931-1.401 | 0.840-1.194 | — | 0.470-1.317 | 0.815-1.478 |
|  |  | 0.502 | 0.147 | 0.136 | 0.626 | 0.194 | 0.445 | **0.057** | 0.444 | 0.904 | 0.942 | 0.469 | 0.573 | 0.203 | 0.984 | — | 0.362 | 0.539 |
|  | rs2598116 | 0.747 | 1.066 | 1.080 | 0.611 | 1.029 | 0.933 | 0.851 | 0.974 | 1.022 | 0.440 | 0.733 | 0.648 | 0.829 | 0.786 | 0.787 | — | 0.876 |
|  |  | 0.390-1.431 | 0.811-1.401 | 0.820-1.422 | 0.382-0.976 | 0.773-1.370 | 0.628-1.387 | 0.464-1.561 | 0.655-1.448 | 0.725-1.442 | 0.156-1.241 | 0.513-1.048 | 0.311-1.350 | 0.416-1.649 | 0.489-1.263 | 0.470-1.317 | — | 0.459-1.671 |
|  |  | 0.379 | 0.648 | 0.584 | **0.039** | 0.844 | 0.732 | 0.602 | 0.897 | 0.900 | 0.121 | **0.089** | 0.247 | 0.592 | 0.320 | 0.362 | — | 0.688 |
|  | rs1802074 | 0.593 | 1.387 | 1.121 | 0.965 | 1.085 | 1.178 | 0.331 | 1.059 | 0.991 | 0.942 | 0.743 | 0.745 | 1.187 | 1.227 | 1.098 | 0.876 | — |
|  |  | 0.271-1.297 | 1.057-1.821 | 0.843-1.492 | 0.573-1.624 | 0.814-1.445 | 0.812-1.709 | 0.157-0.696 | 0.734-1.528 | 0.714-1.374 | 0.442-2.009 | 0.439-1.258 | 0.412-1.346 | 0.846-1.666 | 0.794-1.896 | 0.815-1.478 | 0.459-1.671 | — |
|  |  | 0.190 | **0.018** | 0.432 | 0.893 | 0.580 | 0.388 | **0.004** | 0.760 | 0.956 | 0.878 | 0.269 | 0.330 | 0.320 | 0.356 | 0.539 | 0.688 | — |

Data from logistic regression analyses were represented as OR, 95% CI, and P-value (ONFH vs control).
