# Supplementary material for: 17 variants interaction of Wnt/β-catenin pathway associated with development of osteonecrosis of femoral head in Chinese Han population
Source: Sci Rep. 2024 Mar 27;14:7301. doi: 10.1038/s41598-024-57929-8 (PMC10973331; doi:10.1038/s41598-024-57929-8)
Supplement: Supplementary file 1 — Supplementary Tables. [file 41598_2024_57929_MOESM1_ESM.zip › Supplementary Tables/Supplementary Table 5.docx]

**Supplementary Table 5. Associations of interactions among 17 variants in Wnt/β-catenin pathway with unilateral and bilateral hips lesions of ONFH**

|  |  | **Gsk3β** | | | | | **LRP5** | | | | **EPDR1** | **LOC105375236** | **SFRP4** | | | | | |
| --- | --- | --- | --- | --- | --- | --- | --- | --- | --- | --- | --- | --- | --- | --- | --- | --- | --- | --- |
|  |  | rs2037547 | rs334558 | rs3732361 | rs3755557 | rs6438552 | rs2306862 | rs312778 | rs3736228 | rs556442 | rs16879765 | rs1721400 | rs1052981 | rs1376264 | rs1802073 | rs2084651 | rs2598116 | rs1802074 |
| Gsk3β | rs2037547 | — | 1.357 | 1.610 | 1.254 | 1.755 | 1.553 | 1.276 | 1.563 | 1.719 | — | — | 1.134 | 2.029 | 1.879 | 1.847 | 1.213 | 2.370 |
|  |  | — | 0.640-2.880 | 0.461-5.618 | 0.427-3.684 | 0.426-7.228 | 0.569-4.235 | 0.207-7.850 | 0.573-4.263 | 0.658-4.493 | — | — | 0.453-2.841 | 0.648-6.359 | 0.855-4.129 | 0.898-3.799 | 0.538-2.734 | 0.737-7.620 |
|  |  | — | 0.426 | 0.455 | 0.681 | 0.436 | 0.390 | 0.793 | 0.383 | 0.269 | — | — | 0.789 | 0.225 | 0.116 | **0.096** | 0.641 | 0.148 |
|  | rs334558 | 1.357 | — | 1.008 | 0.856 | 1.039 | 1.045 | 1.092 | 1.088 | 0.907 | 1.106 | 0.938 | 0.801 | 0.926 | 0.927 | 0.951 | 1.189 | 1.169 |
|  |  | 0.640-2.880 | — | 0.835-1.216 | 0.545-1.346 | 0.858-1.259 | 0.750-1.454 | 0.664-1.796 | 0.785-1.509 | 0.677-1.214 | 0.674-1.815 | 0.651-1.350 | 0.562-1.141 | 0.660-1.299 | 0.719-1.196 | 0.752-1.202 | 0.869-1.626 | 0.853-1.601 |
|  |  | 0.426 | — | 0.935 | 0.502 | 0.693 | 0.796 | 0.729 | 0.612 | 0.510 | 0.690 | 0.729 | 0.219 | 0.657 | 0.562 | 0.673 | 0.279 | 0.332 |
|  | rs3732361 | 1.610 | 1.008 | — | 0.825 | 1.040 | 1.232 | 1.039 | 1.232 | 1.015 | 0.867 | 0.939 | 0.803 | 0.883 | 0.881 | 0.905 | 1.275 | 1.275 |
|  |  | 0.461-5.618 | 0.835-1.216 | — | 0.394-1.728 | 0.873-1.238 | 0.875-1.735 | 0.587-1.837 | 0.885-1.715 | 0.768-1.341 | 0.518-1.449 | 0.638-1.380 | 0.554-1.164 | 0.621-1.257 | 0.685-1.134 | 0.711-1.151 | 0.914-1.781 | 0.882-1.844 |
|  |  | 0.455 | 0.935 | — | 0.610 | 0.663 | 0.233 | 0.896 | 0.216 | 0.916 | 0.585 | 0.748 | 0.248 | 0.491 | 0.326 | 0.415 | 0.153 | 0.197 |
|  | rs3755557 | 1.254 | 0.856 | 0.825 | — | 0.797 | 0.456 | 0.620 | 0.442 | 0.377 | 0.699 | 1.274 | 0.647 | 0.784 | 0.708 | 0.776 | 0.934 | 0.548 |
|  |  | 0.427-3.684 | 0.545-1.346 | 0.394-1.728 | — | 0.378-1.678 | 0.222-0.939 | 0.227-1.694 | 0.216-0.906 | 0.191-0.746 | 0.271-1.801 | 0.598-2.716 | 0.361-1.159 | 0.447-1.374 | 0.479-1.047 | 0.530-1.134 | 0.539-1.620 | 0.277-1.084 |
|  |  | 0.681 | 0.502 | 0.610 | — | 0.550 | **0.033** | 0.351 | **0.026** | **0.005** | 0.458 | 0.530 | 0.143 | 0.396 | **0.084** | 0.190 | 0.809 | **0.084** |
|  | rs6438552 | 1.755 | 1.039 | 1.040 | 0.797 | — | 1.180 | 1.221 | 1.181 | 0.959 | 0.925 | 0.908 | 0.893 | 0.951 | 0.934 | 0.940 | 1.242 | 1.265 |
|  |  | 0.426-7.228 | 0.858-1.259 | 0.873-1.238 | 0.378-1.678 | — | 0.823-1.692 | 0.673-2.214 | 0.835-1.670 | 0.719-1.280 | 0.543-1.577 | 0.610-1.353 | 0.614-1.298 | 0.661-1.367 | 0.720-1.210 | 0.733-1.204 | 0.872-1.770 | 0.870-1.839 |
|  |  | 0.436 | 0.693 | 0.663 | 0.550 | — | 0.367 | 0.511 | 0.346 | 0.778 | 0.776 | 0.636 | 0.553 | 0.786 | 0.604 | 0.623 | 0.230 | 0.218 |
| LRP5 | rs2306862 | 1.553 | 1.045 | 1.232 | 0.456 | 1.180 | — | 2.934 | 1.047 | 1.104 | 1.092 | 0.940 | 0.955 | 0.706 | 0.872 | 0.959 | 1.231 | 1.139 |
|  |  | 0.569-4.235 | 0.750-1.454 | 0.875-1.735 | 0.222-0.939 | 0.823-1.692 | — | 0.757-11.375 | 0.781-1.402 | 0.821-1.484 | 0.432-2.758 | 0.503-1.756 | 0.529-1.726 | 0.383-1.301 | 0.595-1.276 | 0.655-1.404 | 0.745-2.034 | 0.712-1.824 |
|  |  | 0.390 | 0.796 | 0.233 | **0.033** | 0.367 | — | 0.120 | 0.761 | 0.513 | 0.853 | 0.845 | 0.880 | 0.264 | 0.479 | 0.831 | 0.417 | 0.586 |
|  | rs312778 | 1.276 | 1.092 | 1.039 | 0.620 | 1.221 | 2.934 | — | 1.859 | 1.424 | 0.856 | 0.947 | 0.559 | 0.948 | 0.878 | 0.600 | 1.637 | 2.993 |
|  |  | 0.207-7.850 | 0.664-1.796 | 0.587-1.837 | 0.227-1.694 | 0.673-2.214 | 0.757-11.375 | — | 0.651-5.312 | 0.687-2.950 | 0.332-2.208 | 0.320-2.809 | 0.194-1.608 | 0.431-2.082 | 0.476-1.617 | 0.306-1.176 | 0.766-3.495 | 0.817-10.959 |
|  |  | 0.793 | 0.729 | 0.896 | 0.351 | 0.511 | 0.120 | — | 0.247 | 0.342 | 0.748 | 0.922 | 0.281 | 0.894 | 0.675 | 0.137 | 0.203 | **0.098** |
|  | rs3736228 | 1.563 | 1.088 | 1.232 | 0.442 | 1.181 | 1.047 | 1.859 | — | 1.155 | 0.956 | 1.039 | 0.912 | 0.844 | 0.920 | 0.992 | 1.233 | 1.292 |
|  |  | 0.573-4.263 | 0.785-1.509 | 0.885-1.715 | 0.216-0.906 | 0.835-1.670 | 0.781-1.402 | 0.651-5.312 | — | 0.855-1.559 | 0.422-2.162 | 0.580-1.860 | 0.518-1.605 | 0.467-1.525 | 0.635-1.333 | 0.685-1.437 | 0.750-2.026 | 0.798-2.094 |
|  |  | 0.383 | 0.612 | 0.216 | **0.026** | 0.346 | 0.761 | 0.247 | — | 0.347 | 0.913 | 0.898 | 0.749 | 0.575 | 0.660 | 0.967 | 0.409 | 0.297 |
|  | rs556442 | 1.719 | 0.907 | 1.015 | 0.377 | 0.959 | 1.104 | 1.424 | 1.155 | — | 1.065 | 1.081 | 0.945 | 0.836 | 0.774 | 0.883 | 1.146 | 1.200 |
|  |  | 0.658-4.493 | 0.677-1.214 | 0.768-1.341 | 0.191-0.746 | 0.719-1.280 | 0.821-1.484 | 0.687-2.950 | 0.855-1.559 | — | 0.553-2.049 | 0.639-1.831 | 0.568-1.573 | 0.496-1.411 | 0.548-1.093 | 0.627-1.243 | 0.754-1.742 | 0.775-1.857 |
|  |  | 0.269 | 0.510 | 0.916 | **0.005** | 0.778 | 0.513 | 0.342 | 0.347 | — | 0.851 | 0.771 | 0.828 | 0.503 | 0.146 | 0.475 | 0.524 | 0.414 |
| EPDR1 | rs16879765 | — | 1.106 | 0.867 | 0.699 | 0.925 | 1.092 | 0.856 | 0.956 | 1.065 | — | 0.969 | 0.989 | 1.089 | 1.042 | 1.142 | 2.364 | 1.132 |
|  |  | — | 0.674-1.815 | 0.518-1.449 | 0.271-1.801 | 0.543-1.577 | 0.432-2.758 | 0.332-2.208 | 0.422-2.162 | 0.553-2.049 | — | 0.348-2.698 | 0.602-1.624 | 0.640-1.852 | 0.691-1.569 | 0.761-1.713 | 0.493-11.343 | 0.468-2.737 |
|  |  | — | 0.690 | 0.585 | 0.458 | 0.776 | 0.853 | 0.748 | 0.913 | 0.851 | — | 0.952 | 0.964 | 0.754 | 0.846 | 0.522 | 0.282 | 0.783 |
| LOC105375236 | rs1721400 | — | 0.938 | 0.939 | 1.274 | 0.908 | 0.940 | 0.947 | 1.039 | 1.081 | 0.969 | — | 0.738 | 1.491 | 1.106 | 1.219 | 1.175 | 1.160 |
|  |  | — | 0.651-1.350 | 0.638-1.380 | 0.598-2.716 | 0.610-1.353 | 0.503-1.756 | 0.320-2.809 | 0.580-1.860 | 0.639-1.831 | 0.348-2.698 | — | 0.303-1.794 | 0.648-3.432 | 0.718-1.705 | 0.738-2.016 | 0.734-1.879 | 0.553-2.436 |
|  |  | — | 0.729 | 0.748 | 0.530 | 0.636 | 0.845 | 0.922 | 0.898 | 0.771 | 0.952 | — | 0.502 | 0.347 | 0.647 | 0.439 | 0.501 | 0.695 |
| SFRP4 | rs1052981 | 1.134 | 0.801 | 0.803 | 0.647 | 0.893 | 0.955 | 0.559 | 0.912 | 0.945 | 0.989 | 0.738 | — | 0.876 | 0.943 | 0.918 | 0.594 | 0.781 |
|  |  | 0.453-2.841 | 0.562-1.141 | 0.554-1.164 | 0.361-1.159 | 0.614-1.298 | 0.529-1.726 | 0.194-1.608 | 0.518-1.605 | 0.568-1.573 | 0.602-1.624 | 0.303-1.794 | — | 0.631-1.217 | 0.666-1.335 | 0.684-1.232 | 0.241-1.463 | 0.380-1.607 |
|  |  | 0.789 | 0.219 | 0.248 | 0.143 | 0.553 | 0.880 | 0.281 | 0.749 | 0.828 | 0.964 | 0.502 | — | 0.431 | 0.742 | 0.568 | 0.257 | 0.502 |
|  | rs1376264 | 2.029 | 0.926 | 0.883 | 0.784 | 0.951 | 0.706 | 0.948 | 0.844 | 0.836 | 1.089 | 1.491 | 0.876 | — | 0.892 | 0.946 | 0.725 | 1.078 |
|  |  | 0.648-6.359 | 0.660-1.299 | 0.621-1.257 | 0.447-1.374 | 0.661-1.367 | 0.383-1.301 | 0.431-2.082 | 0.467-1.525 | 0.496-1.411 | 0.640-1.852 | 0.648-3.432 | 0.631-1.217 | — | 0.634-1.254 | 0.741-1.208 | 0.305-1.719 | 0.720-1.614 |
|  |  | 0.225 | 0.657 | 0.491 | 0.396 | 0.786 | 0.264 | 0.894 | 0.575 | 0.503 | 0.754 | 0.347 | 0.431 | — | 0.510 | 0.657 | 0.465 | 0.714 |
|  | rs1802073 | 1.879 | 0.927 | 0.881 | 0.708 | 0.934 | 0.872 | 0.878 | 0.920 | 0.774 | 1.042 | 1.106 | 0.943 | 0.892 | — | 0.952 | 0.926 | 1.087 |
|  |  | 0.855-4.129 | 0.719-1.196 | 0.685-1.134 | 0.479-1.047 | 0.720-1.210 | 0.595-1.276 | 0.476-1.617 | 0.635-1.333 | 0.548-1.093 | 0.691-1.569 | 0.718-1.705 | 0.666-1.335 | 0.634-1.254 | — | 0.763-1.189 | 0.514-1.669 | 0.640-1.844 |
|  |  | 0.116 | 0.562 | 0.326 | **0.084** | 0.604 | 0.479 | 0.675 | 0.660 | 0.146 | 0.846 | 0.647 | 0.742 | 0.510 | — | 0.665 | 0.799 | 0.758 |
|  | rs2084651 | 1.847 | 0.951 | 0.905 | 0.776 | 0.940 | 0.959 | 0.600 | 0.992 | 0.883 | 1.142 | 1.219 | 0.918 | 0.946 | 0.952 | — | 1.040 | 1.148 |
|  |  | 0.898-3.799 | 0.752-1.202 | 0.711-1.151 | 0.530-1.134 | 0.733-1.204 | 0.655-1.404 | 0.306-1.176 | 0.685-1.437 | 0.627-1.243 | 0.761-1.713 | 0.738-2.016 | 0.684-1.232 | 0.741-1.208 | 0.763-1.189 | — | 0.538-2.014 | 0.802-1.642 |
|  |  | **0.096** | 0.673 | 0.415 | 0.190 | 0.623 | 0.831 | 0.137 | 0.967 | 0.475 | 0.522 | 0.439 | 0.568 | 0.657 | 0.665 | — | 0.906 | 0.451 |
|  | rs2598116 | 1.213 | 1.189 | 1.275 | 0.934 | 1.242 | 1.231 | 1.637 | 1.233 | 1.146 | 2.364 | 1.175 | 0.594 | 0.725 | 0.926 | 1.040 | — | 0.968 |
|  |  | 0.538-2.734 | 0.869-1.626 | 0.914-1.781 | 0.539-1.620 | 0.872-1.770 | 0.745-2.034 | 0.766-3.495 | 0.750-2.026 | 0.754-1.742 | 0.493-11.343 | 0.734-1.879 | 0.241-1.463 | 0.305-1.719 | 0.514-1.669 | 0.538-2.014 | — | 0.412-2.277 |
|  |  | 0.641 | 0.279 | 0.153 | 0.809 | 0.230 | 0.417 | 0.203 | 0.409 | 0.524 | 0.282 | 0.501 | 0.257 | 0.465 | 0.799 | 0.906 | — | 0.941 |
|  | rs1802074 | 2.370 | 1.169 | 1.275 | 0.548 | 1.265 | 1.139 | 2.993 | 1.292 | 1.200 | 1.132 | 1.160 | 0.781 | 1.078 | 1.087 | 1.148 | 0.968 | — |
|  |  | 0.737-7.620 | 0.853-1.601 | 0.882-1.844 | 0.277-1.084 | 0.870-1.839 | 0.712-1.824 | 0.817-10.959 | 0.798-2.094 | 0.775-1.857 | 0.468-2.737 | 0.553-2.436 | 0.380-1.607 | 0.720-1.614 | 0.640-1.844 | 0.802-1.642 | 0.412-2.277 | — |
|  |  | 0.148 | 0.332 | 0.197 | **0.084** | 0.218 | 0.586 | **0.098** | 0.297 | 0.414 | 0.783 | 0.695 | 0.502 | 0.714 | 0.758 | 0.451 | 0.941 | — |

Data from logistic regression analyses were represented as OR, 95% CI, and P-value (bilateral vs unilateral hips).
