# Supplementary material for: 17 variants interaction of Wnt/β-catenin pathway associated with development of osteonecrosis of femoral head in Chinese Han population
Source: Sci Rep. 2024 Mar 27;14:7301. doi: 10.1038/s41598-024-57929-8 (PMC10973331; doi:10.1038/s41598-024-57929-8)
Supplement: Supplementary file 1 — Supplementary Tables. [file 41598_2024_57929_MOESM1_ESM.zip › Supplementary Tables/Supplementary Table 6.docx]

**Supplementary Table 6. Association of paired gene–gene interactions of 17 variants in Wnt/β-catenin pathway with the clinical stages of ONFH.**

|  |  | **Gsk3β** | | | | | **LRP5** | | | | **EPDR1** | **LOC105375236** | **SFRP4** | | | | | |
| --- | --- | --- | --- | --- | --- | --- | --- | --- | --- | --- | --- | --- | --- | --- | --- | --- | --- | --- |
|  |  | rs2037547 | rs334558 | rs3732361 | rs3755557 | rs6438552 | rs2306862 | rs312778 | rs3736228 | rs556442 | rs16879765 | rs1721400 | rs1052981 | rs1376264 | rs1802073 | rs2084651 | rs2598116 | rs1802074 |
| Gsk3β | rs2037547 | — | 1.332 | 2.241 | 0.921 | 1.626 | 1.417 | 2.064 | 1.411 | 1.205 | 0.526 | 0.628 | 1.797 | 1.250 | 0.900 | 0.977 | 0.858 | 1.366 |
|  |  | — | 0.570-3.112 | 0.462-10.879 | 0.314-2.707 | 0.316-8.364 | 0.485-4.139 | 0.225-18.913 | 0.483-4.120 | 0.469-3.095 | 0.031-9.046 | 0.193-2.043 | 0.480-6.731 | 0.392-3.980 | 0.436-1.858 | 0.495-1.927 | 0.381-1.934 | 0.431-4.330 |
|  |  | — | 0.508 | 0.317 | 0.882 | 0.561 | 0.523 | 0.522 | 0.529 | 0.699 | 0.658 | 0.440 | 0.384 | 0.706 | 0.775 | 0.946 | 0.712 | 0.596 |
|  | rs334558 | 1.332 | — | 1.110 | 0.924 | 1.146 | 1.172 | 1.385 | 1.094 | 1.128 | 2.270 | 0.935 | 1.107 | 1.108 | 1.033 | 1.022 | 1.279 | 0.967 |
|  |  | 0.570-3.112 | — | 0.902-1.366 | 0.577-1.479 | 0.924-1.421 | 0.812-1.692 | 0.775-2.478 | 0.766-1.564 | 0.816-1.558 | 1.114-4.628 | 0.640-1.366 | 0.757-1.617 | 0.759-1.618 | 0.788-1.354 | 0.793-1.317 | 0.899-1.820 | 0.694-1.346 |
|  |  | 0.508 | — | 0.323 | 0.741 | 0.215 | 0.397 | 0.272 | 0.621 | 0.466 | **0.024** | 0.729 | 0.600 | 0.595 | 0.814 | 0.865 | 0.171 | 0.841 |
|  | rs3732361 | 2.241 | 1.110 | — | 1.254 | 1.071 | 1.190 | 1.050 | 1.039 | 0.955 | 1.721 | 0.893 | 1.021 | 1.047 | 1.024 | 1.022 | 1.027 | 1.028 |
|  |  | 0.462-10.879 | 0.902-1.366 | — | 0.560-2.810 | 0.884-1.298 | 0.801-1.767 | 0.567-1.945 | 0.719-1.503 | 0.705-1.293 | 0.890-3.328 | 0.600-1.328 | 0.688-1.513 | 0.707-1.549 | 0.781-1.341 | 0.785-1.331 | 0.724-1.459 | 0.697-1.516 |
|  |  | 0.317 | 0.323 | — | 0.582 | 0.485 | 0.390 | 0.877 | 0.837 | 0.766 | 0.107 | 0.575 | 0.919 | 0.820 | 0.866 | 0.869 | 0.880 | 0.889 |
|  | rs3755557 | 0.921 | 0.924 | 1.254 | — | 1.206 | 0.607 | 1.913 | 0.640 | 0.743 | 1.495 | 1.196 | 1.085 | 1.055 | 0.727 | 0.731 | 1.220 | 0.761 |
|  |  | 0.314-2.707 | 0.577-1.479 | 0.560-2.810 | — | 0.537-2.708 | 0.290-1.268 | 0.543-6.739 | 0.309-1.324 | 0.381-1.448 | 0.488-4.579 | 0.522-2.740 | 0.583-2.021 | 0.570-1.952 | 0.488-1.083 | 0.494-1.081 | 0.649-2.293 | 0.385-1.501 |
|  |  | 0.882 | 0.741 | 0.582 | — | 0.650 | 0.184 | 0.312 | 0.229 | 0.382 | 0.481 | 0.673 | 0.797 | 0.864 | 0.117 | 0.116 | 0.537 | 0.430 |
|  | rs6438552 | 1.626 | 1.146 | 1.071 | 1.206 | — | 1.187 | 1.048 | 1.015 | 0.942 | 2.099 | 0.931 | 1.066 | 1.083 | 1.039 | 1.069 | 1.026 | 1.032 |
|  |  | 0.316-8.364 | 0.924-1.421 | 0.884-1.298 | 0.537-2.708 | — | 0.778-1.809 | 0.565-1.943 | 0.687-1.501 | 0.688-1.289 | 0.981-4.492 | 0.618-1.403 | 0.707-1.606 | 0.721-1.627 | 0.785-1.376 | 0.810-1.411 | 0.707-1.491 | 0.695-1.531 |
|  |  | 0.561 | 0.215 | 0.485 | 0.650 | — | 0.426 | 0.883 | 0.939 | 0.709 | **0.056** | 0.733 | 0.761 | 0.701 | 0.790 | 0.638 | 0.891 | 0.878 |
| LRP5 | rs2306862 | 1.417 | 1.172 | 1.190 | 0.607 | 1.187 | — | 0.966 | 1.174 | 1.300 | 2.337 | 0.943 | 1.588 | 1.673 | 1.014 | 1.297 | 0.949 | 1.252 |
|  |  | 0.485-4.139 | 0.812-1.692 | 0.801-1.767 | 0.290-1.268 | 0.778-1.809 | — | 0.272-3.426 | 0.824-1.672 | 0.892-1.895 | 0.672-8.131 | 0.497-1.789 | 0.741-3.404 | 0.777-3.601 | 0.671-1.532 | 0.833-2.019 | 0.559-1.612 | 0.707-2.216 |
|  |  | 0.523 | 0.397 | 0.390 | 0.184 | 0.426 | — | 0.957 | 0.373 | 0.172 | 0.182 | 0.857 | 0.235 | 0.188 | 0.948 | 0.250 | 0.847 | 0.441 |
|  | rs312778 | 2.064 | 1.385 | 1.050 | 1.913 | 1.048 | 0.966 | — | 0.867 | 1.140 | 1.082 | 1.257 | 0.912 | 1.084 | 1.489 | 1.057 | 0.935 | 1.227 |
|  |  | 0.225-18.913 | 0.775-2.478 | 0.567-1.945 | 0.543-6.739 | 0.565-1.943 | 0.272-3.426 | — | 0.302-2.495 | 0.531-2.451 | 0.395-2.964 | 0.394-4.010 | 0.332-2.506 | 0.458-2.562 | 0.734-3.019 | 0.535-2.090 | 0.453-1.930 | 0.397-3.793 |
|  |  | 0.522 | 0.272 | 0.877 | 0.312 | 0.883 | 0.957 | — | 0.792 | 0.736 | 0.878 | 0.700 | 0.858 | 0.855 | 0.270 | 0.873 | 0.855 | 0.723 |
|  | rs3736228 | 1.411 | 1.094 | 1.039 | 0.640 | 1.015 | 1.174 | 0.867 | — | 1.232 | 1.559 | 0.839 | 1.239 | 1.214 | 0.881 | 1.069 | 0.992 | 1.215 |
|  |  | 0.483-4.120 | 0.766-1.564 | 0.719-1.503 | 0.309-1.324 | 0.687-1.501 | 0.824-1.672 | 0.302-2.495 | — | 0.854-1.779 | 0.587-4.138 | 0.463-1.521 | 0.640-2.400 | 0.607-2.427 | 0.594-1.307 | 0.708-1.614 | 0.584-1.684 | 0.679-2.172 |
|  |  | 0.529 | 0.621 | 0.837 | 0.229 | 0.939 | 0.373 | 0.792 | — | 0.265 | 0.373 | 0.564 | 0.525 | 0.584 | 0.529 | 0.752 | 0.975 | 0.512 |
|  | rs556442 | 1.205 | 1.128 | 0.955 | 0.743 | 0.942 | 1.300 | 1.140 | 1.232 | — | 2.331 | 0.662 | 1.770 | 1.667 | 0.908 | 1.194 | 0.890 | 1.185 |
|  |  | 0.469-3.095 | 0.816-1.558 | 0.705-1.293 | 0.381-1.448 | 0.688-1.289 | 0.892-1.895 | 0.531-2.451 | 0.854-1.779 | — | 0.803-6.764 | 0.389-1.128 | 0.830-3.773 | 0.846-3.286 | 0.628-1.314 | 0.810-1.759 | 0.572-1.385 | 0.703-1.995 |
|  |  | 0.699 | 0.466 | 0.766 | 0.382 | 0.709 | 0.172 | 0.736 | 0.265 | — | 0.120 | 0.129 | 0.139 | 0.140 | 0.609 | 0.370 | 0.605 | 0.524 |
| EPDR1 | rs16879765 | 0.526 | 2.270 | 1.721 | 1.495 | 2.099 | 2.337 | 1.082 | 1.559 | 2.331 | — | 2.153 | 0.965 | 1.206 | 1.420 | 1.450 | 3.376 | 2.441 |
|  |  | 0.031-9.046 | 1.114-4.628 | 0.890-3.328 | 0.488-4.579 | 0.981-4.492 | 0.672-8.131 | 0.395-2.964 | 0.587-4.138 | 0.803-6.764 | — | 0.527-8.786 | 0.574-1.623 | 0.647-2.247 | 0.873-2.310 | 0.874-2.407 | 0.442-25.802 | 0.620-9.611 |
|  |  | 0.658 | **0.024** | 0.107 | 0.481 | **0.056** | 0.182 | 0.878 | 0.373 | 0.120 | — | 0.285 | 0.893 | 0.556 | 0.157 | 0.150 | 0.241 | 0.202 |
| LOC105375236 | rs1721400 | 0.628 | 0.935 | 0.893 | 1.196 | 0.931 | 0.943 | 1.257 | 0.839 | 0.662 | 2.153 | — | 0.717 | 0.892 | 1.110 | 0.984 | 0.799 | 1.539 |
|  |  | 0.193-2.043 | 0.640-1.366 | 0.600-1.328 | 0.522-2.740 | 0.618-1.403 | 0.497-1.789 | 0.394-4.010 | 0.463-1.521 | 0.389-1.128 | 0.527-8.786 | — | 0.290-1.773 | 0.391-2.036 | 0.695-1.770 | 0.577-1.675 | 0.503-1.271 | 0.636-3.726 |
|  |  | 0.440 | 0.729 | 0.575 | 0.673 | 0.733 | 0.857 | 0.700 | 0.564 | 0.129 | 0.285 | — | 0.471 | 0.786 | 0.663 | 0.951 | 0.344 | 0.339 |
| SFRP4 | rs1052981 | 1.797 | 1.107 | 1.021 | 1.085 | 1.066 | 1.588 | 0.912 | 1.239 | 1.770 | 0.965 | 0.717 | — | 0.940 | 1.073 | 1.042 | 0.744 | 1.201 |
|  |  | 0.480-6.731 | 0.757-1.617 | 0.688-1.513 | 0.583-2.021 | 0.707-1.606 | 0.741-3.404 | 0.332-2.506 | 0.640-2.400 | 0.830-3.773 | 0.574-1.623 | 0.290-1.773 | — | 0.653-1.351 | 0.723-1.593 | 0.745-1.457 | 0.287-1.927 | 0.508-2.840 |
|  |  | 0.384 | 0.600 | 0.919 | 0.797 | 0.761 | 0.235 | 0.858 | 0.525 | 0.139 | 0.893 | 0.471 | — | 0.737 | 0.726 | 0.810 | 0.542 | 0.676 |
|  | rs1376264 | 1.250 | 1.108 | 1.047 | 1.055 | 1.083 | 1.673 | 1.084 | 1.214 | 1.667 | 1.206 | 0.892 | 0.940 | — | 1.028 | 1.123 | 0.563 | 1.365 |
|  |  | 0.392-3.980 | 0.759-1.618 | 0.707-1.549 | 0.570-1.952 | 0.721-1.627 | 0.777-3.601 | 0.458-2.562 | 0.607-2.427 | 0.846-3.286 | 0.647-2.247 | 0.391-2.036 | 0.653-1.351 | — | 0.701-1.506 | 0.847-1.489 | 0.230-1.380 | 0.829-2.249 |
|  |  | 0.706 | 0.595 | 0.820 | 0.864 | 0.701 | 0.188 | 0.855 | 0.584 | 0.140 | 0.556 | 0.786 | 0.737 | — | 0.888 | 0.419 | 0.209 | 0.221 |
|  | rs1802073 | 0.900 | 1.033 | 1.024 | 0.727 | 1.039 | 1.014 | 1.489 | 0.881 | 0.908 | 1.420 | 1.110 | 1.073 | 1.028 | — | 0.862 | 1.267 | 0.745 |
|  |  | 0.436-1.858 | 0.788-1.354 | 0.781-1.341 | 0.488-1.083 | 0.785-1.376 | 0.671-1.532 | 0.734-3.019 | 0.594-1.307 | 0.628-1.314 | 0.873-2.310 | 0.695-1.770 | 0.723-1.593 | 0.701-1.506 | — | 0.680-1.093 | 0.658-2.439 | 0.427-1.298 |
|  |  | 0.775 | 0.814 | 0.866 | 0.117 | 0.790 | 0.948 | 0.270 | 0.529 | 0.609 | 0.157 | 0.663 | 0.726 | 0.888 | — | 0.220 | 0.479 | 0.299 |
|  | rs2084651 | 0.977 | 1.022 | 1.022 | 0.731 | 1.069 | 1.297 | 1.057 | 1.069 | 1.194 | 1.450 | 0.984 | 1.042 | 1.123 | 0.862 | — | 1.161 | 1.006 |
|  |  | 0.495-1.927 | 0.793-1.317 | 0.785-1.331 | 0.494-1.081 | 0.810-1.411 | 0.833-2.019 | 0.535-2.090 | 0.708-1.614 | 0.810-1.759 | 0.874-2.407 | 0.577-1.675 | 0.745-1.457 | 0.847-1.489 | 0.680-1.093 | — | 0.562-2.400 | 0.688-1.473 |
|  |  | 0.946 | 0.865 | 0.869 | 0.116 | 0.638 | 0.250 | 0.873 | 0.752 | 0.370 | 0.150 | 0.951 | 0.810 | 0.419 | 0.220 | — | 0.687 | 0.974 |
|  | rs2598116 | 0.858 | 1.279 | 1.027 | 1.220 | 1.026 | 0.949 | 0.935 | 0.992 | 0.890 | 3.376 | 0.799 | 0.744 | 0.563 | 1.267 | 1.161 | — | 1.066 |
|  |  | 0.381-1.934 | 0.899-1.820 | 0.724-1.459 | 0.649-2.293 | 0.707-1.491 | 0.559-1.612 | 0.453-1.930 | 0.584-1.684 | 0.572-1.385 | 0.442-25.802 | 0.503-1.271 | 0.287-1.927 | 0.230-1.380 | 0.658-2.439 | 0.562-2.400 | — | 0.433-2.627 |
|  |  | 0.712 | 0.171 | 0.880 | 0.537 | 0.891 | 0.847 | 0.855 | 0.975 | 0.605 | 0.241 | 0.344 | 0.542 | 0.209 | 0.479 | 0.687 | — | 0.889 |
|  | rs1802074 | 1.366 | 0.967 | 1.028 | 0.761 | 1.032 | 1.252 | 1.227 | 1.215 | 1.185 | 2.441 | 1.539 | 1.201 | 1.365 | 0.745 | 1.006 | 1.066 | — |
|  |  | 0.431-4.330 | 0.694-1.346 | 0.697-1.516 | 0.385-1.501 | 0.695-1.531 | 0.707-2.216 | 0.397-3.793 | 0.679-2.172 | 0.703-1.995 | 0.620-9.611 | 0.636-3.726 | 0.508-2.840 | 0.829-2.249 | 0.427-1.298 | 0.688-1.473 | 0.433-2.627 | — |
|  |  | 0.596 | 0.841 | 0.889 | 0.430 | 0.878 | 0.441 | 0.723 | 0.512 | 0.524 | 0.202 | 0.339 | 0.676 | 0.221 | 0.299 | 0.974 | 0.889 | — |

Data from logistic regression analyses were represented as OR, 95% CI, and p-value (stage IV vs. stage III).
