# Supplementary material for: 17 variants interaction of Wnt/β-catenin pathway associated with development of osteonecrosis of femoral head in Chinese Han population
Source: Sci Rep. 2024 Mar 27;14:7301. doi: 10.1038/s41598-024-57929-8 (PMC10973331; doi:10.1038/s41598-024-57929-8)
Supplement: Supplementary file 1 — Supplementary Tables. [file 41598_2024_57929_MOESM1_ESM.zip › Supplementary Tables/Supplementary Table 7.docx]

**Supplementary Table 7. Associations of multidimensional interactions among 17 variants in Wnt/β-catenin pathway with ONFH risk.**

| **Model** | **Training**  **balanced**  **accuracy** | **Testing**  **balanced**  **accuracy** | **SIGN Test (P)** | **CVconsistency** |
| --- | --- | --- | --- | --- |
| rs334558 | 0.5594 | 0.5241 | 8 (***0.05***) | 9/10 |
| rs334558-rs3732361 | 0.5975 | 0.5315 | 6 (0.38) | 5/10 |
| rs334558-rs1052981-rs1376264 | 0.6382 | 0.5422 | 8 (***0.05***) | 4/10 |
| rs334558-rs6438552-rs1802073-rs2084651 | 0.6904 | 0.5264 | 7 (0.17) | 3/10 |
| rs334558-rs6438552-rs556442-rs1802073-rs2084651 | 0.7463 | 0.5396 | 8 (***0.05***) | 6/10 |
| rs334558-rs6438552-rs556442-rs1721400-rs1052981-rs1802073 | 0.8033 | 0.5475 | 6 (0.38) | 6/10 |
| rs334558-rs6438552-rs556442-rs1721400-rs1052981-rs1802073-rs2084651 | 0.8561 | 0.5939 | 8 (***0.05***) | 10/10 |
| rs334558-rs6438552-rs312778-rs556442-rs1721400-rs1052981-rs1802073-rs2084651 | 0.8941 | 0.5355 | 5 (0.62) | 5/10 |
| rs334558-rs3755557-rs6438552-rs312778-rs556442-rs1721400-rs1052981-rs1802073-rs2084651 | 0.9252 | 0.5094 | 7 (0.17) | 3/10 |

P: the interactions among variants.
